# RUG3 and ATM synergistically regulate the alternative splicing of mitochondrial *nad2* and the DNA damage response in *Arabidopsis thaliana*

**DOI:** 10.1038/srep43897

**Published:** 2017-03-06

**Authors:** Chao Su, Hongtao Zhao, Yankun Zhao, Hongtao Ji, Youning Wang, Liya Zhi, Xia Li

**Affiliations:** 1 State Key Laboratory of Agricultural Microbiology, College of Plant Science and Technology Huazhong Agricultural University, Wuhan 430070, P.R. China; 2 Center for Agricultural Research Resources, Institute of Genetics and Developmental Biology, Chinese Academy of Sciences, Hebei 050021, P.R. China; 3 College of Life Sciences, Hebei Normal University, Hebei 050024, P.R. China; 4 Shijiazhuang Academy of Agricultural and Forestry Sciences, Hebei 050041, P.R. China

## Abstract

The root apical meristem (RAM) determines both RAM activity and the growth of roots. Plant roots are constantly exposed to adverse environmental stresses that can cause DNA damage or cell cycle arrest in the RAM; however, the mechanism linking root meristematic activity and RAM size to the DNA damage response (DDR) is unclear. Here, we demonstrate that a loss of function in *RCC1/UVR8/GEF-Like 3 (RUG3*) substantially augmented the DDR and produced a cell cycle arrest in the RAM in *rug3* mutant, leading to root growth retardation. Furthermore, the mutation of *RUG3* caused increased intracellular reactive oxygen species (ROS) levels, and ROS scavengers improved the observed cell cycle arrest and reduced RAM activity level in *rug3* plants. Most importantly, we detected a physical interaction between RUG3 and ataxia telangiectasia mutated (ATM), a key regulator of the DDR, suggesting that they synergistically modulated the alternative splicing of *nad2*. Our findings reveal a novel synergistic effect of RUG3 and ATM on the regulation of mitochondrial function, redox homeostasis, and the DDR in the RAM, and outline a protective mechanism for DNA damage repair and the restoration of mitochondrial function that involves RUG3-mediated mitochondrial retrograde signaling and the activation of an ATM-mediated DDR pathway.

Primary growth in plant roots is governed largely by the root apical meristem (RAM), which harbors stem cells that continuously divide asymmetrically to generate new stem cells and daughter cells that undergo additional rounds of cell division^1^,^2^,^3^. The meristematic activity of the RAM is dynamically and precisely controlled through the integration of developmental and environmental signals^4^. Under optimal conditions, cell division in the meristematic zone remains active and roots grow normally and indeterminately. However, the activity of the RAM may be reduced by adverse environmental changes^5^, leading to a cell cycle arrest. Under conditions of severe stress, meristematic cells in the root completely lose their ability to divide, resulting in a permanent cell cycle arrest and determinate root growth^6^,^7^. Although some genes that regulate this cell cycle arrest and/or the activity of the RAM have been identified, the upstream signals and signaling cascades that cause the cell cycle arrest and reduced meristematic activity in the RAM in response to environmental stresses remain unclear.

Reactive oxygen species (ROS) are important regulators of meristematic activity in the RAM^8^,^9^. Compelling evidence indicates that controlled ROS levels and cellular redox homeostasis are essential for root growth. For example, hydrogen peroxide (H_2_O_2_) accumulation in the root apex results in retarded root growth^8^, whereas the increased expression of antioxidant genes known to control H_2_O_2_ levels (e.g., glutathione [GSH]) is sufficient for RAM organization^10^. It has also been shown that the regulatory role of GSH in root growth is due to its redox buffering capacity^11^,^12^. Genetic analyses of the genes encoding NADPH-dependent thioredoxin reductase A (*NTRA*), NADPH-dependent thioredoxin reductase B (*NTRB*), and cinnamyl alcohol dehydrogenase homolog 2 (*CAD2*), which mediate thioredoxin reduction and GSH synthesis, have confirmed the important role of cellular redox status in RAM activity^13^. Based on these studies and a functional analysis of NADPH oxidase, it was proposed that NADPH oxidase plays a crucial role in ROS generation and redox regulation of root growth^13^. ROS levels and cellular redox homeostasis appear to be controlled genetically and specific ROS levels may be required for the regulation of plant root development^8^,^12^,^13^.

Despite the existence of a complex network controlling ROS production and scavenging in cells, environmental stress and genotoxic agents can induce excessive ROS accumulation leading to the oxidation of proteins, lipids, and DNA, and even cell death^14^,^15^,^16^. DNA oxidation can cause DNA single- or double-strand breaks, resulting in genomic instability. DNA damage can trigger the DNA damage response (DDR) through ataxia telangiectasia mutated (ATM)- or ATM and Rad 3-related (ATR)-mediated pathways depending on the presence of DSBs or single-strand breaks^17^,^18^. ATM- and ATR-mediated signaling cascades are known to activate the G2 checkpoint kinase WEE1, which negatively regulates the G2-to-M phase transition, resulting in a cell cycle arrest and the induction of downstream effectors that enable cells to repair DNA damage before proceeding to mitosis^19^. In mammals, oxidative DNA damage and the DDR have been well characterized. Recently, the coupling of oxidative DNA damage and activation of the DDR with the activation of a G2/M cell cycle arrest was demonstrated using a mutant defective in two ROS-detoxifying enzymes, APX1 (cytosol) and CAT2 (peroxisome)^20^. In that study, it was demonstrated that the extra nuclear detoxification of ROS is essential to protect DNA from oxidative stress. However, the relationship between ROS and the DDR in plants, especially in the RAM, is unknown.

Mitochondria are a major source of ROS, and mitochondrial ROS (mROS) are essential for activation of the DDR in mammalian and yeast cells^21^,^22^,^23^,^24^. Although mitochondrial complexes I, II, and III have been shown to be direct sources of mROS in plants^25^,^26^,^27^, several recent reports have identified complex I as the main source of mROS, which are associated with meristem activity and root growth^8^,^28^,^29^. The genes responsible for complex I function include *ABA Overly sensitive 6 (ABO6*) and *ABA Overly sensitive 8 (ABO8*), which encode a DEXH-box RNA helicase and a P-type pentatricopeptide repeat (PPR) domain protein, respectively^8^,^29^. ABO6 regulates *nad2* alternative splicing^29^, while ABO8 is responsible for the splicing of *nad4* pre-mRNA^8^. Both *nad2* and *nad4* encode the subunit of mitochondrial NAD(P)H dehydrogenase and function in stability of mitochondrial complex I and ROS homeostasis, respectively^8^,^30^. Interestingly, both ABO6 and ABO8 affect ROS accumulation in the RAM, cell cycle progression, and root growth rates under normal conditions and in response to ABA, implying a key role for mROS in cell cycle progression and the meristematic activity of the RAM in plants. However, whether mROS induce the DDR in the RAM, and how mitochondria and the nucleus communicate with each other to prevent oxidative damage to DNA are unknown.

To elucidate the role of regulator of chromosome condensation 1 (RCC1) family proteins in modulating root meristem activity, we performed a genetic screen using mutants defective in 24 *RCC1* family genes^30^, Two mutants carrying mutations in *RCC1/UVR8/GEF-Like 3 (RUG3*) that showed delayed root growth were selected^30^. Given that RUG3 regulates *nad2* pre-mRNA splicing and mitochondrial complex I biogenesis^30^, we were interested in the role of RUG3 in the regulation of mROS and the DDR during root cell cycle progression, and in its role in mitochondrial retrograde signaling in plants. Here, we show that the mutation of RUG3 caused delayed root growth and plant development. Intriguingly, we also found that RUG3 interacts directly with ATM to synergistically regulate *nad2* pre-mRNA splicing, mROS accumulation, the DDR, and cell cycle progression. Our results indicate a novel role for ATM in modulating mitochondrial function and redox homeostasis, and they reveal a new mechanism leading to the activation of mitochondrial retrograde signaling in the ATM-mediated DDR and the maintenance of mitochondrial function in RAM homeostasis and activity.

## Results

### A loss of function in *RUG3* results in short roots and delayed development

**Figure 1:**
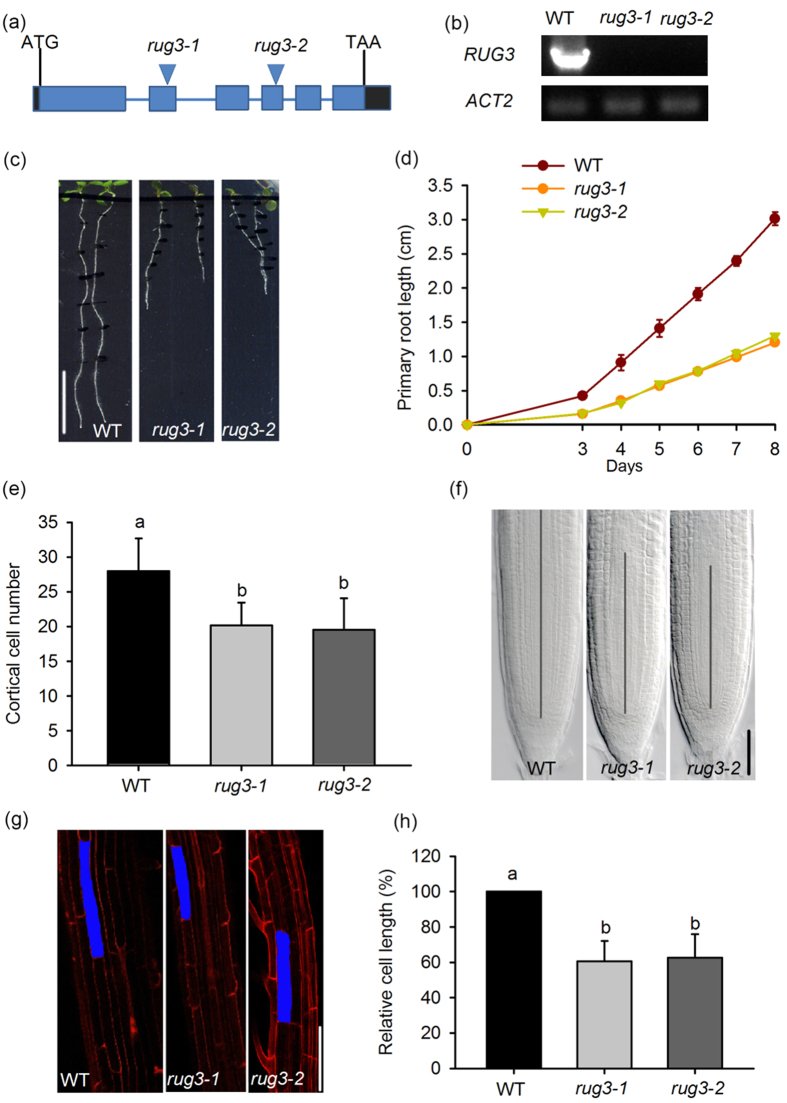
The mutation of *RUG3* reduces the size of the root meristem. (**a**) Diagram of the T-DNA insertion lines, the closed rectangles represent exons; the lines between the exons denote introns. (**b**) RT-PCR analysis of the expression of *RUG3* in wild type, *rug3-1*, and *rug3-2. ACT2* was used as an internal control. The full-length gel can be find in supplementary information file the full-length gel for (**b**). (**c**) Phenotypes of WT, *rug3-1*, and *rug3-2* seedlings at 8 days after germination (DAG). Three-day-old seedlings grown on MS medium were grown vertically on MS medium for another 5 days before being photographed. Bar = 1 cm. (**d**) Quantitative analysis of primary root length at different time points. Three biological repeats were done with similar results. The data shown are means ± standard deviation (SD) (n = 30). (**e**) Cortical cell number in the meristematic zone in WT, *rug3-1* and *rug3-2* plants at 6 DAG. The data shown are means ± SD (n = 30). Different letters represent significant differences. (**f**) Comparison of root meristem size in WT, *rug3-1* and *rug3-2* plants at 6 DAG. The vertical lines indicate the root meristem length. Bar = 100 μm. (**g**) Mature root cell length in WT, *rug3-1* and *rug3-2* plants at 6 DAG. The images were taken after Propidium Iodide (PI) staining. Bar = 60 μm. (**h**) Quantitative analysis of the mature root cell lengths in WT, *rug3-1* and *rug3-2* young seedlings at 6 DAG. The data shown are means + SD (n = 30). Different letters represent significant differences (Student-Newman-Kuels test, *P* < 0.05).

To identify novel genetic loci that regulate root growth, we performed a phenotypic analysis of loss-of-function mutants of 24 genes encoding RCC1 family proteins by sowing the seeds on Murashige and Skoog (MS) medium for 8 days. The mutants with mutations in *RUG3* (At5g60870) caused by T-DNA insertions (Supplementary Fig. S1) and undetectable levels of *RUG3* mRNA showed severe root growth defects (Fig. 1a–c). The mutants (SALK_092071 and N336814) we used in this study were previously designated as *rug3-1* and *rug3-2*, respectively^30^.

A previous study showed that a loss of function in *RUG3* caused retarded development in *Arabidopsis* including the root growth defects^30^; however, a detailed description of the influence of *RUG3* mutations on root growth and development is lacking. To further investigate the role of *RUG3* in plant root development, we conducted a systematic phenotypic analysis of *rug3* mutants. As shown in Fig. 1c–h, *rug3-1* and *rug3-2* mutant plants grew much more slowly than did wild-type (WT) roots after radical emergence, and the primary root length in both *rug3* mutants was reduced by approximately 55% at 8 days after stratification (Fig. 1c,d). Notably, the size of the RAM in the mutant primary roots was markedly reduced, and the average cortical cell number in the RAM of the *rug3* plants was dramatically decreased compared with that in wild type (Fig. 1e,f). Furthermore, the average length of mature root cells in the *rug3* mutants was dramatically decreased compared with that in wild type (Fig. 1g,h). These results suggest that *RUG3* modulates both RAM activity and root cell elongation in *Arabidopsis*.

In addition to root growth, both *rug3* mutants showed delayed development. For example, the mutants exhibited late germination and cotyledon greening compared with WT plants (Supplementary Fig. S2a–c). At the seedling stage, the *rug3* plants were much smaller than wild type (Supplementary Fig. S2d), and the flowering time of the mutants was slightly delayed (Supplementary Fig. S2e; Supplementary Fig. S3a,b). As a result, the life span of the mutants was dramatically extended compared with that of wild type. Also, the stature of the *rug3* mutants was comparable to that of wild type at the mature stage. Together, these data indicate that *RUG3* also plays a pivotal role in vegetative growth and the phase transition from vegetative to reproductive growth.

Since these mutant alleles of *RUG3* produced the same developmental defects, we assessed whether the mutant phenotypes were caused by a loss of function in RUG3. As expected, overexpression of *RUG3* under the control of the CaMV35S promoter in a *rug3-1* background completely restored all of the developmental phenotypes of the *rug3-1 mutant* (Supplementary Fig. S4a–e), confirming the aforementioned role of *RUG3* in plant development. Thereafter, we used *rug3-1* to examine the function of *RUG3* in root growth and plant development.

### The *rug3* mutant show increased cell cycle arrest at the G2/M checkpoint

**Figure 2:**
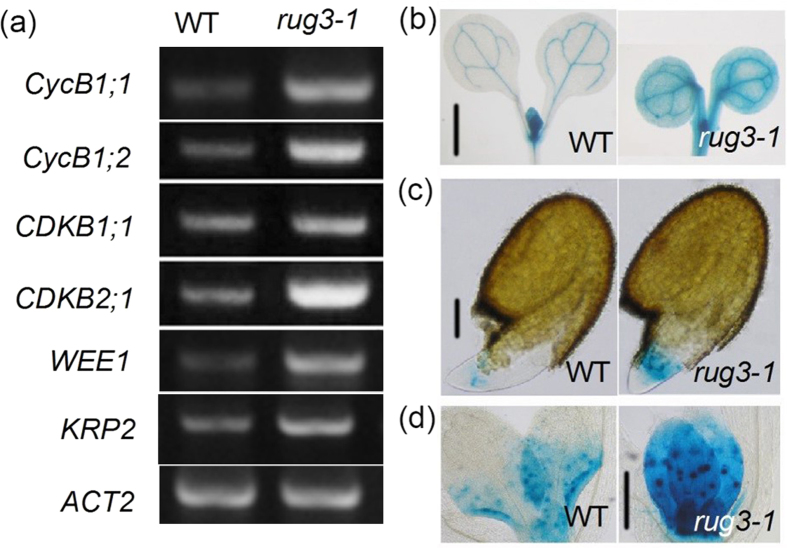
The mutation of *RUG3* results in a cell cycle arrest at G2/M phase. (**a**) Expression of cell cycle-related genes in WT and *rug3-1* plants*. ACT2* was used as an internal control. The full-length gels can be find in supplementary information file the full-length gel for (**a**). (**b**) *WEE1pro*::*GUS* expression in WT (left) and *rug3-1* (right) plants after grown on MS medium for 6 days and 9 days respectively. Bar = 3 mm. (**c**) *CycB1;1pro*::*GUS* expression in WT (left) and *rug3-1* (right) plants after grown on MS medium for 2 days and 3 days respectively. Bar = 150 μm. (**d**) *CycB1;1pro*::*GUS* expression in WT (left) and *rug3-1* (right) plants after grown on MS medium for 7 days and 10 days respectively. Bar = 3 mm.

To assess whether the short roots observed in *rug3-1* plants were due to a defect in cell cycle regulation, we analyzed the expression of the marker gene of cell cycle in *rug3-1* mutant plants. Our expression analysis show that the expression of *KRP2*, which is a main negative regulator of G1/S checkpoint^31^, was not changed (Supplementary Fig. S4), and the expression of *CDKB1;1*, which is specifically activated during early S phase and M phase^32^ was also not dramatically altered in *rug3-1* mutant plants (Fig. 2a; Supplementary Fig. S5). By contrast, the expression of *CycB1;1, WEE1* and *CDKB2;1*, which activate the G2/M checkpoint^19^,^33^,^34^,^35^ are upregulated (Fig. 2a; Supplementary Fig. S5). To confirm the result, we generated *rug3-1* lines expressing *WEE1pro::GUS* and *CycB1;1pro*::*GUS* assays confirmed the upregulation of *WEE1* and *CycB1;1* transcription in the *rug3-1* mutant background. GUS assays confirmed the upregulation of *WEE1* transcription in the *rug3-1* mutant background (Fig. 2b). The GUS assays also revealed *CycB1;1pro::GUS* expression in the meristematic tissues of germinating WT seedlings; in sharp contrast, *CycB1;1pro::GUS* expression was substantially increased in *rug3-1* roots and shoots during early development (Fig. 2c,d). Taken together, these results indicate that *RUG3* may function as a negative regulator of G2/M checkpoint activation.

### The mutation of *RUG3* exacerbates DNA damage

**Figure 3:**
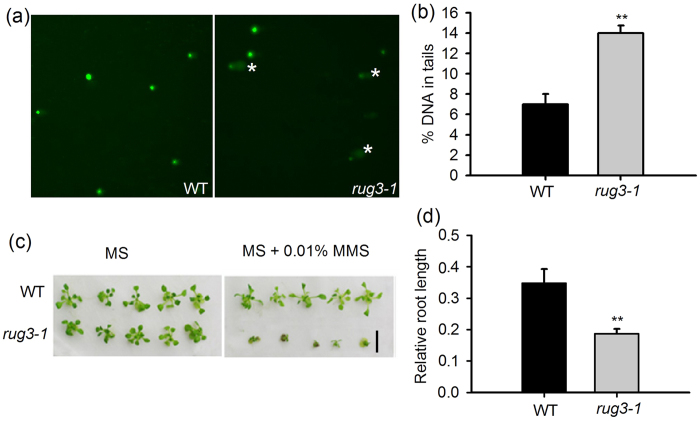
The mutant *rug3-1* is responsive to chemically induced DNA damage. (**a**) Analysis of DNA damage in WT and *rug3-1* plants by a comet assay. Nine-day-old seedlings grown on MS medium were used for nuclear extraction. *Indicates the comet tails in the *rug3-1* plants. (**b**) Quantitative analysis of the DNA in tails of the WT and *rug3-1* plants shown in (**a**). (Student’s *t*-test, where **P < 0.01). Three biological repeats were done with similar results. The data shown are means ± SD. (**c**) The mutant *rug3-1* showed greater sensitivity to MMS than WT plants. Nine-day-old seedlings grown on MS medium were transferred to MS medium containing 0 or 0.01% MMS for another 15 days before photographed. Bar = 6 mm. (**d**) Quantitative analysis of root length in WT and *rug3-1* plants treated with MMS. Seeds were grown vertically on MS medium containing 0 or 0.01% MMS for 15 days. (Student’s *t*-test, where **P < 0.01).

The G2/M checkpoint is also known as the G2/M DNA damage checkpoint, ensures that cells repair damage to their DNA before entering mitosis^19^,^36^. Based on the role of *RUG3* in controlling the G2/M checkpoint, we speculated that *RUG3* is involved in regulating the DDR in *Arabidopsis*. If this were the case, *rug3-1* mutants should exhibit excessive DNA damage. To test this, we performed a comet assay to evaluate the DNA damage in 9-day-old seedlings grown under normal conditions. As expected, the percentage of DNA in the tail of *rug3-1* cells was significantly higher than that in wild type (Fig. 3a,b), pointing to a role for *RUG3* in the DDR at the G2/M checkpoint. Next, we examined the response of *rug3* plants to the DSB-inducing agent methyl methanesulfonate (MMS; 0.01%) and found that *rug3-1* was hypersensitive to MMS (Fig. 3c). Treated *rug3-1* seedlings displayed greater root and shoot growth inhibition than did WT seedlings (Fig. 3c,d). These results suggest an important role for *RUG3* in the DSB repair response. To further assess whether *RUG3* mediates DNA replication, we analyzed the sensitivity of *rug3-1* plants to a chemical inducer of DNA damage, the ribonucleotide reductase inhibitor hydroxyurea (HU). The result shows that the sensitivity of the *rug3-1* plants to HU was comparable to that of the wild type (Supplementary Fig. S6), indicating that the RUG3 does not mediate the DNA replication stress in Arabidopsis.

Since the *rug3-1* mutant show hypersensitive to DSB-inducing agent MMS, we assessed whether the mutant phenotypes were caused by a loss of function in *RUG3*. As expected, the transgenic line *Re-4* can completely restored the greening rate phenotypes of the *rug3-1* mutant and partial restoration the short root phenotypes (Supplementary Fig. S7), confirming the aforementioned role of *RUG3* in DNA damage response.

### *RUG3* is ubiquitously expressed and may be repressed by MMS

**Figure 4:**
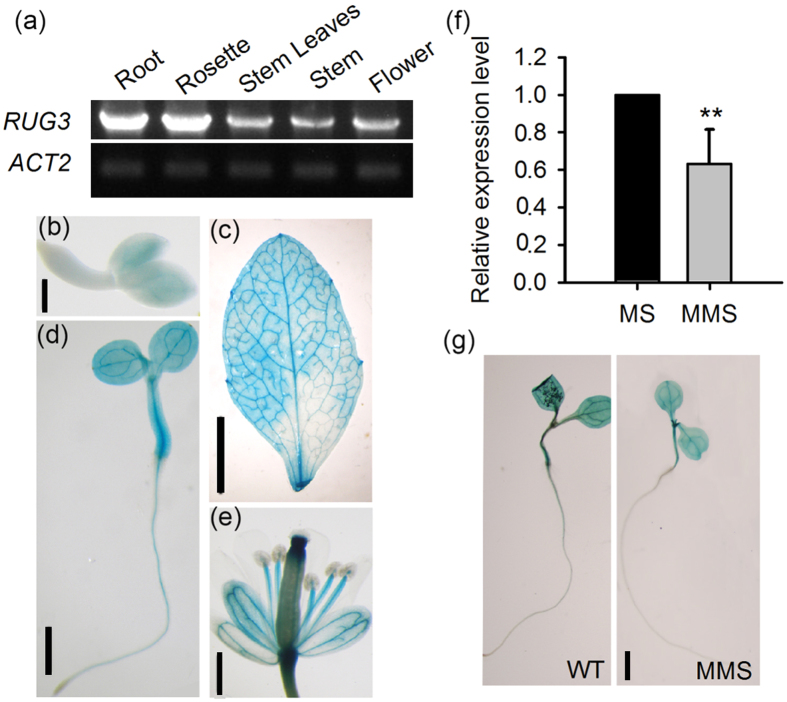
*RUG3* is ubiquitously expressed and repressed by DNA damage reagents. (**a**) RT-PCR analysis the *RUG3* expression in various tissues. *ACT2* was used as an internal control. The full-length gels can be find in supplementary information file the full-length gel for (**a**). (**b**–**f**) Histochemical staining for *RUG3pro::GUS* in various tissues at germination stage (**b**), Bar = 150 μm), grown on MS medium for 3 days (**c**), Bar = 4 mm), 6 days (**d**), Bar = 5 mm), rosette leaves grown for 30 days (**e**), Bar = 1 cm) and flowers (**f**), Bar = 2 mm). (**g**) The expression of *RUG3* under MMS treatment. Seven-day-old seedlings grown on MS medium were transferred to liquid MS medium containing 0.01% MMS for another 12 h (Bar = 5 mm). Three biological replicates were done with similar results. The data shown are means + SD. Different letters represent significant differences (**Student-Newman-Kuels test, *P* < 0.01). (**h**) GUS staining analysis of *RUG3* expression in plants treated with MMS. Seven-day-old *RUG3pro::GUS* transgenic seedlings grown on MS medium were transferred to liquid MS medium containing 0.01% MMS for another 12 h. Three biological replicates were done with similar results. Bar = 4 mm.

Although it has been reported that RUG3 is a mitochondrial protein based on the bioinformatic prediction of an N-terminal transit peptide targeting the protein to mitochondria and the demonstrated subcellular localization of N-terminus_RUG3_-GFP in *Arabidopsis* protoplasts^30^, information about the expression pattern of *RUG3* and the subcellular localization of RUG3 is lacking. To address this, we first analyzed the expression pattern of *RUG3*. Semi-quantitative PCR showed that *RUG3* was expressed in multiple tissues, including the roots and rosette leaves of young seedlings and in the flowers and siliques of mature plants, with higher expression levels in the roots and rosette leaves of young seedlings (Fig. 4a). We next generated transgenic lines expressing *RUG3pro::GUS* for histochemical staining; our results confirmed the ubiquitous expression of *RUG3. RUG3* was preferentially expressed in the vascular tissues of the various organs and tissues tested (Fig. 4b–e), the preferential expression in the vasculature fits to the fact that phloem cells contain most mitochondria^37^.

To check whether *RUG3* is responsive to DNA damage and DNA replication stress, we analyzed the transcription level of *RUG3* in response to MMS treatment. As shown in Fig. 4f, the mRNA level of *RUG3* was markedly reduced by MMS. GUS staining confirmed that *RUG3* transcription was repressed by MMS (Fig. 4g).

### RUG3 is localized to mitochondria and interact with ATM

**Figure 5:**
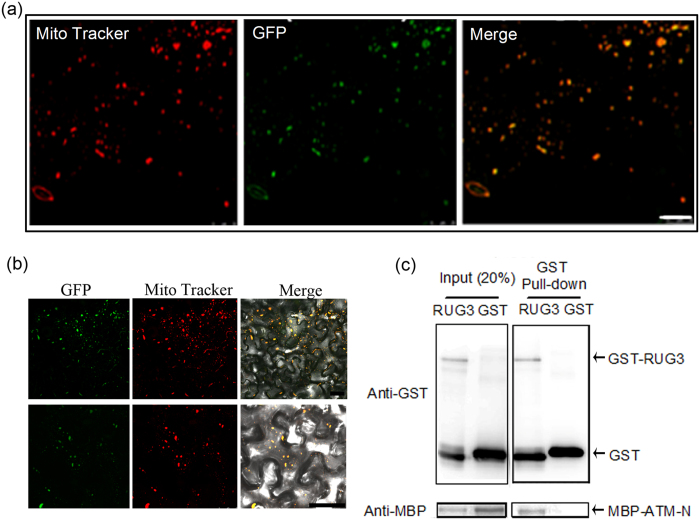
RUG3 is localized to mitochondria and interacts with ATM. (**a**) Subcellular localization of RUG3 protein. Construct harboring *RUG3pro::RUG3-GFP* was transformed into *N. benthamiana* leaves; RUG3-GFP fluorescence was observed after 2 days of cultivation. The mitochondrial localization of RUG3 was confirmed using MitoTracker. Bar = 25 μm. (**b**) RUG3 interacted directly with ATM in a BiFC assay. Constructs harboring RUG3-YN and ATM-N-YC were transformed into *N. benthamiana* leaves and YFP fluorescence was observed in the epidermal cells after 2 days of cultivation. The mitochondrial localization of the interaction was confirmed by MitoTracker staining. (up: many cells in the field of vision; bottom: only one cell in the field of vision), Bar = 25 μm. (**c**) A pull-down assay showed that RUG3 interacted directly with ATM. Equal amounts of the purified ATM-N-MBP fusion protein were added to tubes containing GSH-Sepharose resin plus GST or RUG3-GST, respectively. Anti-MBP antibodies were used to detect the RUG3-ATM complex. The full-length gels can be find in supplementary information file the full-length gel for (**c**).

To further characterize the subcellular localization of full-length RUG3, we expressed RUG3-GFP under the control of the 35 S promoter in *Nicotiana benthamiana* leaf cells. As shown in Fig 5a, RUG3-GFP was detected in mitochondria. The observed mitochondrial localization of RUG3 is consistent with previous data obtained using the N-terminus of RUG3^30^, and it confirms the role of RUG3 in modulating mitochondrial function. RCC1 is a guanine nucleotide exchange factor (GEF) for the small GTP-binding protein Ran^38^,^39^. Although we find RUG3 is localized to mitochondria, we still wondered whether RUG3 has the similar roles. As we expected, the results showed that, like UVR8 (UV RESISTANCE LOCUS 8), RUG3 did not have detectable GEF activity (Supplementary Fig. S8).

ATM is a key upstream regulator of the DDR, and it was recently demonstrated to localize to and function in mitochondria in human cells^40^,^41^. ATM is a large protein composed of 3845 amino acids; thus, it was difficult to express the full-length protein. Since ATM contains an N-terminal PWWP domain, which is involved in protein-protein interactions^42^, we used the N-terminal region of ATM (300 amino acids; ATMn) in our experiments. To further characterize the subcellular localization of Arabidopsis ATM, we expressed the N-terminal sequence of ATM fused with GFP under the control of the 35 S promoter in *Nicotiana benthamiana* leaf cells. Localization of ATMn-GFP in mitochondria was confirmed by Mitotracker staining (Supplementary Fig. S9).

Because RUG3 is colocalized with ATM in mitochondria and has a similar role in the DDR, we speculated that RUG3 and ATM regulate mitochondrial function and cellular responses to DNA damage via a direct or indirect interaction. To test this possibility, we performed protein-protein interaction assays. Intriguingly, a bimolecular fluorescence complementation (BiFC) assay showed that RUG3 interacted directly with ATMn in *N. benthamiana* leaf cells (Fig. 5b). This interaction was confirmed by a pull-down assay (Fig. 5c). Thus, RUG3 and ATM may work together to modulate mitochondrial function and the DDR.

### ATM and RUG3 synergistically regulate the DNA damage stress response

**Figure 6:**
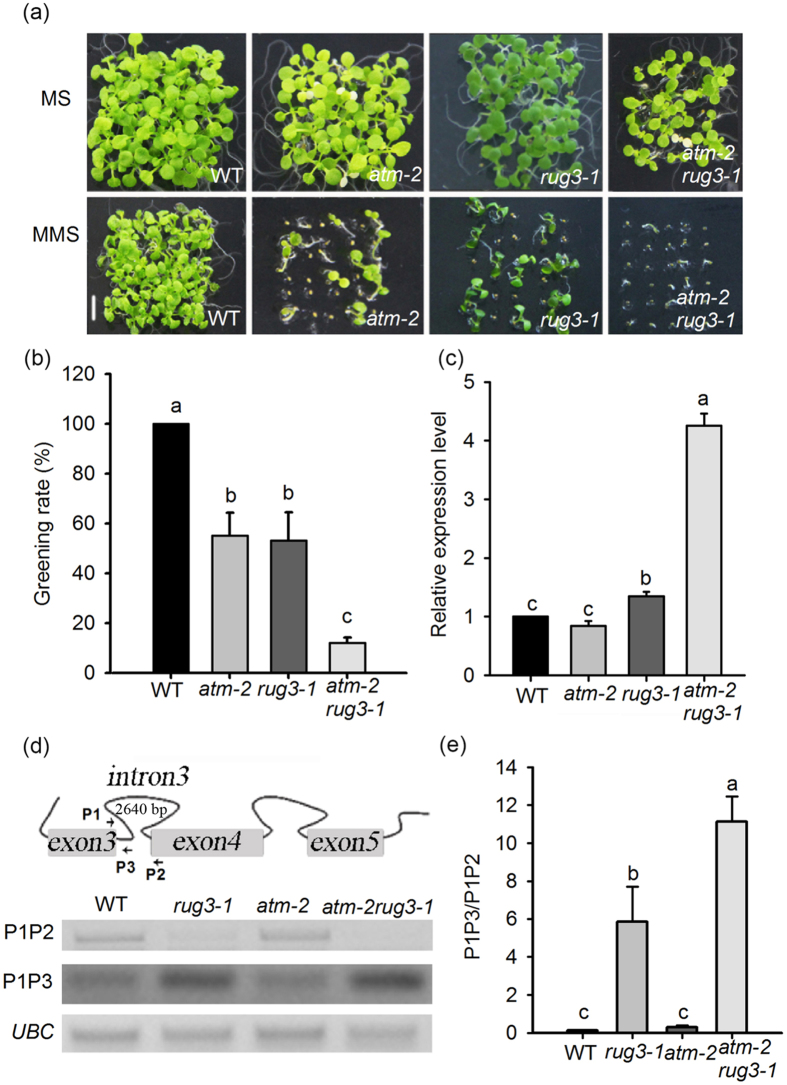
Genetic analysis between *RUG3* and *ATM*. (**a**) Phenotypic analysis of WT, *rug3-1, atm-2*, and *atm-2rug3-1* plants grown on MS medium containing 0 or 0.01% MMS. The pictures were taken at 15 DAG. Bar = 6 mm. (**b**) Quantitative analysis of the greening rate on MS medium containing 0 or 0.01% MMS. Three biological replicates were done with similar results. The data shown are means ± SD. Different letters represent significant differences (Student-Newman-Kuels test, *P* < 0.05). (**c**) Fifteen-day-old seedlings grown on MS medium containing 0.01% MMS were used for analysis. Three biological replicates were done with similar results. The data shown are means ± SD. Different letters represent significant differences. (Student-Newman-Kuels test, *P* < 0.05). (**d**) RT-PCR detect the ratio of intron 3 (the length of intron 3 is 2640 bp) retention transcript and normal transcript level in WT, *rug3-1, atm-2* and *atm-2rug3-1*. P1, P2 and P3 indicate the primers used in the RT-PCR. The full-length gels can be find in supplementary information file the full-length gel for (**d**). (**e**) The relative grey intensities normalized with *UBC* from (**d**) were calculated by Image J software. The data shown are means ± SD. Different letters represent significant differences (Student-Newman-Kuels test, *P* < 0.05).

ATM is a key upstream regulator of the DDR, and then we analyzed the genetic relationship between RUG3 and ATM in response to DNA DSBs in *atm-2rug3-1* double mutant plants produced by a genetic cross. The sensitivity of *rug3-1, atm-2*^43^, and *atm-2rug3-1* double mutant plants to MMS stress was evaluated. When germinated on medium supplemented with 0.01% MMS, *rug3-1* and *atm-2* showed greater sensitivity than WT plants to MMS treatment (Fig. 6a,b; Supplementary Fig. S10). The percentage of germinating *rug3-1* or *atm-2* seedlings that turned green was markedly reduced by about 50% (Fig. 6b). By sharp contrast, MMS sensitivity was exacerbated in germinating *atm-2rug3-1* double mutant plants, and the greening rate dropped sharply to only 12% (Fig. 6b). It is possible that RUG3 and ATM play a synergistic role in regulating the DDR.

### ATM enhances the regulation of *nad2* splicing by RUG3

It has been reported that RUG3 regulates *nad2* pre-mRNA splicing^30^. Given that ATM and RUG3 synergistically regulate the DDR in plants, we speculated that ATM co-regulates the alternative splicing of *nad2* pre-mRNA. If this is the case, *atm-2* mutant plants should have abnormal *nad2* splicing efficiency and *atm-2rug3-1* mutant plants should exhibit even greater defects in *nad2 s*plicing. To test this, we analyzed the level of *nad2* mRNA containing intron 3 in *rug3-1, atm-2*, and *atm-2rug3-1* double mutant plants following MMS (0.01%) treatment. As shown in Fig. 6c, the amount of *nad2* mRNA containing intron 3 was significantly increased in *rug3-1* mutant, consistent with previous data^30^. Unexpectedly, *nad2* splicing in the *atm-2* mutant was comparable to that in wild type. However, to our surprise, the retention of intron 3 in *nad2* mRNA was elevated in the *atm-2rug3-1* double mutant plants. To rule out the possibility that increased retention of intron 3 in *nad2* mRNA is derived from an overall increase in mitochondrial transcripts of *nad2*, we performed RT-PCR to detect both the unspliced and spliced *nad2* transcripts under MMS treatment and calculated the ratio of unspliced transcripts to spliced transcripts. As expected, the unspliced and spliced *nad2* were increased and decreased in *rug3-1* and *atm-2rug3-1* double mutant plants, respectively (Fig. 6d). As a result, the ratio of unspliced transcript to spliced transcript in *atm-2rug3-1* double mutant is significantly higher than *rug3-1* (Fig. 6e). These data indicate that ATM and RUG3 synergistically regulate alternative splicing of *nad2* pre-mRNA.

### A loss of function in both RUG3 and ATM causes ROS overproduction

**Figure 7:**
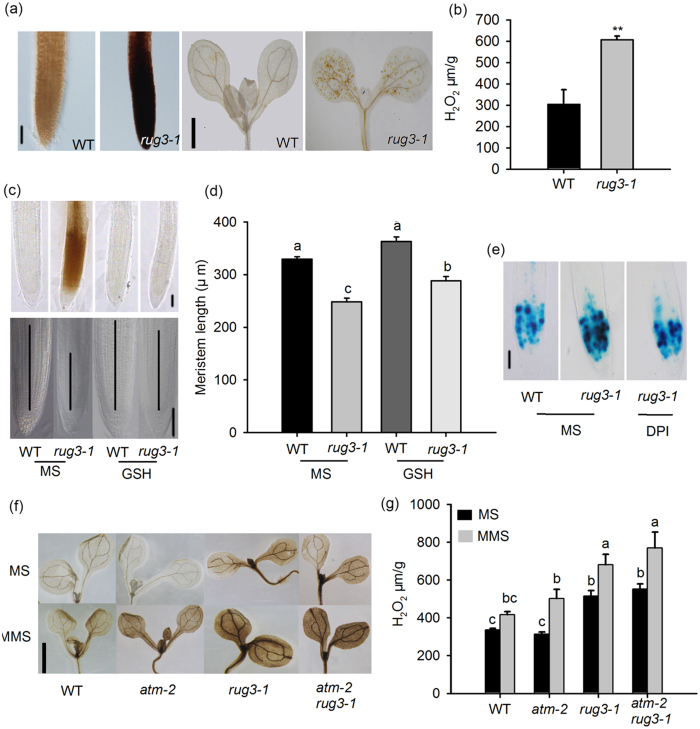
The mutant *rug3-1* accumulates more ROS than WT plants. (**a**) Analysis of H_2_O_2_ accumulation in WT and *rug3-1* plants by DAB staining. Seedlings grown on MS medium were analyzed 7 DAG. Bar = 125 μm (left); Bar = 6 mm (right). (**b**) Quantitative analysis of H_2_O_2_ production in WT and *rug3-1* plants. Seedlings grown on MS medium were analyzed at 7 DAG using an Amplex Red Hydrogen Peroxide/Peroxidase Assay Kit. (Student’s *t*-test, where **P < 0.01). (**c**) GSH reduced ROS accumulation and increased the meristem size in *rug3-1* plants. Seedlings grown on MS medium for 7 days then use 500 μM GSH treatment another 24 h before DAB staining (top); seedlings grown on MS medium containing 0 or 300 μM GSH, the root meristem were analyzed at 7 DAG (bottom). Bar = 125 μm. (**d**) Quantitative data of (**c**). Three biological replicates were done with similar results. The data shown are means ± SD (n = 30). Different letters represent significant differences (Student-Newman-Kuels test, *P* < 0.05). (**e**) *CycB1;1pro::GUS* expression was decreased in *rug3-1* plants treated with DPI. Seven-day-old seedlings grown on MS were transferred to liquid MS medium containing 0 or 0.1 μM DPI for another 10 h before staining. Bar = 125 μm. (**f**) Analysis of the H_2_O_2_ level in WT, *rug3-1, atm-2*, and *rug3-1atm-2* plants treated with MMS. Seven-day-old seedlings grown on MS medium were transferred to liquid MS medium containing 0 or 0.01% MMS and treated for another 12 h before DAB staining. Bar = 6 mm. (**g**) Quantitative analysis of H_2_O_2_ level in the indicated plants. Seven-day-old seedlings grown on MS medium were transferred to liquid MS medium containing 0 or 0.01% MMS and treated for another 12 h; whole plants were examined for H_2_O_2_ production with an Amplex Red Hydrogen Peroxide/Peroxidase Assay Kit. Different letters represent significant differences (Student-Newman-Kuels test, *P* < 0.05).

The gene *nad2* encodes a subunit of mitochondrial complex I, which is the main source of mROS. Since RUG3 and ATM synergistically regulate *nad2* splicing and the DDR, we hypothesized that the increased DDR and cell cycle arrest in the RAM may be due to elevated ROS levels caused by abnormal *nad2* splicing and complex I functionality. We therefore analyzed the role of *RUG3* in modulating ROS levels and the ROS responses of plants. 3,3′-Diaminobenzidine (DAB) staining showed that 7-day-old *rug3-1* mutant plants had substantially increased H_2_O_2_ levels in the RAM and cotyledons under normal conditions compared to wild type (Fig. 7a,b). The high level of ROS accumulation in *rug3-1* was confirmed by dichloro-dihydro-fluorescein diacetate (DCFH-DA) staining (Supplementary Fig. S11), suggesting a role for *RUG3* in maintaining ROS homeostasis.

To assess whether the elevated ROS level in the *rug3-1* mutant causes the DDR and cell cycle arrest in the RAM, we tested the effect of a scavenging agent on the ROS content in *rug3-1* mutant plants. DAB staining clearly showed that the addition of GSH to the growth medium (MS medium) almost completely reversed the high level of ROS in the RAM and largely restored the RAM phenotypes of *rug3-1* mutant plants (Fig. 7c,d). We also used the ROS production inhibitor diphenyleneiodonium (DPI) to analyze that whether reduced ROS production in mitochondria would release the cell cycle arrest defect seen in *rug3*. Following treatment with DPI for 10 h, the *CycB1;1pro::GUS* signal in *rug3* was substantially reduced to the level in wild type (Fig. 7e). These data confirm that RUG3 modulates cell cycle progression in the RAM by maintaining ROS homeostasis.

Next, we tested whether RUG3 and ATM co-regulate mROS accumulation in response to MMS treatment. As shown in Fig. 7f and g, in the presence of MMS, *atm-2* plants accumulated more H_2_O_2_ than did wild type. The level of H_2_O_2_ in the *rug3-1* mutants was higher than that in wild type and increased significantly in response to MMS treatment. The level of H_2_O_2_ in the *atm-2rug3-1* double mutant plants was similar to that in the *rug3-1* mutant with or without MMS treatment. However, further phenotypic analysis showed that the two single mutants and double mutant exhibited different levels of sensitivity to H_2_O_2_ treatment compared with wild type (Supplementary Fig. S12); *atm-2* showed the least sensitivity to H_2_O_2_, *rug3-1* was in the middle, and the *atm-2rug3-1* double mutant showed the greatest sensitivity to H_2_O_2_. Together, these results suggest that ATM does not affect ROS accumulation in plants under normal conditions, but that it modulates ROS accumulation and sensitivity by working together with RUG3 in the DDR.

## Discussion

The growth and development of plants, which are sessile organisms, occur mainly postembryonically and are affected by both developmental and environmental factors. One of the most important questions in plant biology is how meristematic activity and meristem tissue homeostasis are controlled in apical meristems, which determine shoot and root growth rates and the overall architecture of plants. In the RAM, how the meristematic activity of stem cells and their daughter cells is regulated is not precisely known. In particular, the mechanism by which RAM size and activity are modulated in response to the DNA damage caused by abiotic stress remains elusive. RUG3 was previously shown to regulate *nad2* pre-mRNA splicing and complex I biogenesis^30^. In the present study, we found that RUG3 is an upstream regulator of ROS, which trigger retrograde signaling from mitochondria to the nucleus in response to oxidative stress and regulate both the DDR and cell cycle progression in the RAM. We also uncovered a novel mechanism by which ATM interacts with RUG3 in mitochondria to synergistically regulate the alternative splicing of *nad2* pre-mRNA, mitochondrial function, oxidative metabolism, the DDR, and RAM activity.

In human cells, RCC1 acts as a guanine nucleotide exchange factor (GEF) for Ran GTPase; it also has chromatin and DNA binding activity in the nucleus, enabling it to regulate gene expression^38^,^39^,^44^. In plants, there are 24 genes encoding RCC1 family proteins; among them, UVR8 and TCF1 (Tolerant to Chilling and Freezing 1) have been well characterized and both proteins mediate plant response to environmental conditions^45^,^46^,^47^,^48^,^49^. In cells irradiated with ultraviolet B (UVB) light, UVR8 moves from the cytosol to the nucleus, where it activates a signaling cascade that enables the cell to respond to the exposure^46^. When plants are exposed low temperature, TCF1 modulates lignin contents of the stressed cells and plant freezing tolerance through a chromatic-based regulatory network^49^. *RUG3* encodes an RCC1 family protein that contains seven RCC1 tandem repeats and has strong similarity to UVR8. Unexpectedly, RUG3 contains mitochondrial targeting sequences (MTS); both a partial protein containing the MTS^30^ and full-length RUG3 driven by the native promoter were shown to localize to mitochondria (Fig. 5a), suggesting a novel role for RUG3 in mitochondrial function. Indeed, it has previously been shown that RUG3 is specifically required for complex I biogenesis; it regulates the splicing of intron 3 from *nad2* pre-mRNA molecules^30^. Our results also show that, like UVR8, RUG3 does not have detectable GEF activity (Supplementary Fig. S8). Since no DNA or RNA binding activity has been detected for RUG3, it is unclear how RUG3 mediates *nad2* pre-mRNA splicing. It may be involved in *nad2* pre-mRNA splicing through an interaction with ABO5, a PPR protein that can bind RNA and which is required for the splicing of intron 3 from *nad2* pre-mRNA^28^, or through an interaction with other proteins (e.g., mitochondrial transcription termination factor 15 [mTERF15]) that specifically modulate intron 3 splicing from *nad2* pre-mRNA^50^.

A previous study showed that a loss of function in RUG3 resulted in global changes in transcription, including the transcription of genes that function in mitochondria, protein metabolism, and cell organization^30^. However, how RUG3 regulates gene expression on a global scale is unknown. In this study, we produced several lines of evidence to support the hypothesis that RUG3 is an upstream regulator of mitochondrial retrograde signaling and that it controls cell cycle progression in the RAM by modulating ROS production and the DDR. First, we showed that RUG3 is a positive regulator of meristematic activity in the RAM, and that a loss of function in RUG3 resulted in short roots (Fig. 1c–h). Second, we demonstrated that RUG3 is required for cell cycle progression, and that the mutation of RUG3 caused a cell cycle arrest (Fig. 2). Third, we demonstrated that the cell cycle arrest mediated by RUG3 was due to activation of the DDR. The mutation of RUG3 resulted in the increased expression of DDR markers (e.g., *BRCA1* and *RAD51*) and downstream effectors (e.g., *WEE1*) that control the G2/M cell cycle arrest checkpoint (Supplementary Fig. S5; Supplementary Fig. S13). Finally, we showed that RUG3 plays an important role in ROS metabolism. Nonspecific and targeted scavenging of ROS restored the ROS levels and cell cycle progression in a loss-of-function RUG3 mutant. This is not surprising because mitochondrial complex I is the main site of ROS production^8^,^28^,^29^. The overproduction of ROS can cause DNA oxidation and damage^40^,^51^; however, mROS are a key mitochondrial signal that triggers mitochondrial retrograde signaling, which reprograms the expression of nuclear genes and maintains mitochondrial function (Chandel 2014). Notably, previous studies showed that RUG3 regulates the expression of several genes encoding proteins that are essential for mitochondrial function (e.g., *TOM6, TIM13*, and *TIM2*2)^30^. These data suggest a critical role for RUG3 in the regulation of retrograde signaling from mitochondria to the nucleus and in the maintenance of normal mitochondrial functioning. In addition to transcriptional changes, ROS may cause the oxidation of proteins associated with many other biological processes. For example, oxidative stress induces the oxidation of ATM, which is able to activate ATM in the absence of DNA damage^52^.

In mammals, ATM not only activates the DDR and cell cycle arrest, it also modulates mROS levels and mitochondrial functioning^40^,^41^. It has been shown that in mitochondria ATM phosphorylates BH3-interacting domain death agonist (BID), which induces oxidative stress and a cell cycle arrest, as well as the quiescence/renewal of hematopoietic stem cells^41^. However, there are no reports on the mitochondrial localization or function of ATM in plants. In this study, we preliminary found that the ATM is able to interact physically with RUG3 in mitochondria (Fig. 5b,c). Intriguingly, ATM and RUG3 had a synergistic effect on *nad2* pre-mRNA alternative splicing (Fig. 6c–e) and subsequent ROS homeostasis (Fig. 7f,g) and the DDR (Fig. 6a,b). Our data suggest that RUG3 and ATM function as a rheostat to maintain ROS homeostasis by regulating *nad2* pre-mRNA alternative splicing, which is required for proper mitochondrial organization and complex I activity. In this model, RUG3 promotes normal *nad2* pre-mRNA alternative splicing to ensure normal mitochondrial architecture and basal ROS levels under normal conditions; in response to the DDR, the repression of RUG3 results in decreased splicing efficiency, mitochondrial complex I dysfunction, and increased ROS levels. ROS, as mitochondrial retrograde signals, enter the nucleus to activate the ATM-dependent DDR. ATM is also translocated to mitochondria where it attenuates the abnormal alternative splicing of *nad2* pre-mRNA by complexing with RUG3, and in doing so it helps maintain mitochondrial function and ROS homeostasis. To the best of our knowledge, this is the first report on the role of ATM in mitochondrial *nad2* pre-mRNA splicing. However, the mechanism underlying ATM-mediated *nad2* pre-mRNA splicing remains unclear. Recently, it was demonstrated in human cells that ATM targets the core spliceosome in response to DNA damage to regulate genome-wide alternative splicing with a preferential role in intron retention during the DDR^53^. It will be of great interest to determine whether ATM specifically regulates *nad2* pre-mRNA splicing or whether it regulates the alternative splicing of multiple mitochondrial pre-mRNAs in response to DNA damage. Defining the exact mechanism underlying the ATM- and RUG3-mediated maintenance of mitochondrial function and ROS homeostasis in response to DNA damage will further enhance our understanding of the DDR and the regulation of tissue homeostasis in the RAM.

## Methods

### Plant materials and growth conditions

*Arabidopsis thaliana* ecotype Columbia-0 was used in this study. Seeds were surface-sterilized with 50% (v/v) commercial bleach for 5 min, followed by five rinses with sterilized water, and then grown on MS medium (M519; PhytoTechnology Laboratories, Shawnee Mission, KS) containing 1% (w/v) sucrose and 0.8% agar. After 2 days at 4 °C, the seeds were germinated at 23 °C under a 16/8 h light/dark photoperiod.

The marker lines and mutants used in this work are *rug3-1, rug3-2, atm-2, CycB1;1pro::GUS* and *WEE1pro::GUS*. The T-DNA insertion lines SALK_092071 (*rug3-1*; T-DNA inserted at 740 bp downstream of translation position) and SALK_006953 (*atm-2*; T-DNA inserted at 27477 bp of *ATM* genomic DNA) were obtained from the Arabidopsis Biological Resource Center (Columbus, OH), while the T-DNA insertion line N335814 (*rug3-2*; T-DNA inserted at 1430 bp downstream of translation position) was obtained from the Nottingham Arabidopsis Stock Centre (Nottinghamshire, UK).

### Germination, greening and flowering time assay

For phenotypic analysis, MS medium was supplemented with 1% sucrose and with or without MMS. Seeds were surface-sterilized and sown on the plates. The plates were stratified at 4 °C for 2 days then put into the incubator at 23 °C under a 16/8 h light/dark photoperiod. For germination assay the elongated radicles were counted every 24 h. For greening assay the cotyledon greening were counted every 24 h. For flowering time assay, the rate of leaf initiation, the rosette leaf number, the days to see the visible bud and the days of first flower bloom were analyzed under long-day condition (16 h of light/8 h of dark).

### Gene expression analysis

Total RNA was extracted from plant samples using Trizol reagent (Tiangen Biotech [Beijing] Co. Ltd., Beijing, China). First-strand cDNA was synthesized from the total RNA using a FastQuant RT Kit (TransGen Biotech Co., Ltd., Beijing, China). Semi-quantitative RT-PCR and qRT-PCR were done as described previously^5^. *ACT2* was used as an internal control; three independent replicates were performed per experiment. The gene-specific primers used for *WEE1, CycB1;1, CDKB2;1*, and *NAD2-intro3* are listed in Supplementary Table S1.

### Analysis of *nad2* transcript splicing

Measurement of the expression of intron 3 of *nad2* and the splicing of *nad2* by Semi-quantitative RT-PCR in plants treated with MMS. Fifteen-day-old seedlings grown on MS medium containing 0.01% MMS were used for analysis. The relative grey intensities normalized with *UBC* were calculated by the Image J software. The gene-specific primers used are listed in Supplementary Table S1.

### GUS staining assay

The promoter of *RUG3* was cloned into pCAMBIA1391 at the *Eco*RI-*Bam*HI sites to generate the construct containing *RUG3pro*::*GUS. RUG3pro*::*GUS* was then transformed into *Arabidopsis* by the floral dip method. Histochemical staining was performed as described previously^49^.

### Construction of *35S::RUG3-GFP, RUG3pro::RUG3-GFP* and *35S::ATMn-GFP*

For *35S::RUG3-GFP* construction, the coding sequence of *RUG3* was amplified by PCR and the product was digested for insertion into the binary vector pCAMBIA1300 at the *Xba*I-*Bam*HI sites. For *RUG3pro::RUG3-GFP* construction, the promoter of RUG3 (1416 bp) and the coding region of *RUG3* were amplified by PCR, and the products were digested with *Pst*I-*Xba*I and *Xba*I-*Bam*HI, respectively, and then were inserted into the binary vector (pCAMBIA1300) sequentially. For *35 S::ATMn-GFP* construction, the coding sequence of the *ATM* N-terminal sequence (300 amino acids; ATMn) amplified by PCR was cloned to pGW-B6 through the gateway system. The gene-specific primers used for the construction are listed in Supplementary Table S1.

### Subcellular localization analysis

The constructs of *RUG3pro::RUG3-GFP* and *35S::ATMn-GFP* were used for subcellular localization analysis. *Agrobacterium* GV3101 cells containing the constructs were cultured overnight, and then adjusted to OD_600_ = 0.3-0.4 using 10 mM MgCl_2_. The inoculum was injected into the leaves of 4-week-old *N. benthamiana* plants. At 2 days after injection, GFP fluorescence was observed with a Leica SP8 confocal microscope (Jena, Germany) from the epidermal cells of leaves. Mitochondria were stained with MitoTracker (Invitrogen, Carlsbad, CA) for colocalization analysis.

### GEF activity assays

GEF activity assay was performed as described in^54^. Briefly, RCC1, RUG3, and human Ran were expressed in *Escherichia coli* as fusions with GST. The Ran clone was provided by Dr. Murray Stewart (Medical Research Council Laboratory for Molecular Biology, Cambridge, U.K.). Assays of guanine nucleotide exchange activity were performed by using [^3^H]GDP to load 30 pmol GST-Ran and subsequent incubation with 0.5 nM recombinant RCC1 or RUG3 for 3 min. The exchange activity was calculated. The GEF assays were repeated four times.

### Yeast two-hybrid assays

The *RUG3* coding region and *RANs* coding region were amplified by PCR and cloned in pGADT7 and pGBKT7 respectively. Plasmid DNA of bait and prey constructs were transformed into the *S. cerevisiae* strain Y190. Individual transformants were streaked on plates containing a synthetic, minimal (SD) medium lacking tryptophan and leucine and grown for 24 h. Yeast cells were transferred onto a filter paper, and β-galactosidase (β-gal) filter assays were performed as described by Zhu *et al*.^55^.

### Measurement of ROS in plants

For the DCFH-DA staining assay, root tips from 7-day-old seedlings were incubated in darkness for 30 min in a solution containing phosphate-buffered saline (PBS; pH 6.0) and 50 μM DCFH-DA. They were then washed three times with PBS (pH 6.0) to remove the excess DCFH-DA. Fluorescence was detected with a Leica SP8 confocal microscope.

An Amplex Red Hydrogen Peroxide/Peroxidase Assay Kit (Invitrogen) was used to measure H_2_O_2_ production as described by Shin *et al*.^56^.

For DAB staining to detect H_2_O_2_, seven-day-old seedlings were incubated in 0.1 mg/mL DAB (Sigma-Aldrich Corp., St. Louis, MO) dissolved in ddH_2_O (pH 5.0) for 8 h, and then in 75% ethanol at 65 °C for 15 min; after three washes, the roots were photographed.

### Comet assay

A comet assay was performed using a Comet Assay Kit (4250-050-K; Trevigen, Gaithersburg, MD). The comets were visualized by staining with SYBR Green I, and then photographed using a Leica SP8 confocal microscope. Each experimental point is represented by the mean value from three independent experiments. For each material at least 50 nucleuses were photographed, then the data were analyzed by using Casp_1.2.3b1 software.

### BiFC Assay

The binary vectors used in this experiment were pEarlygate201-YN and pEarlygate202-YC^57^,^58^. The coding sequence of *RUG3* was cloned into pEarlygate201-YN to generate RUG3-YFP^N^ while the ATM N-terminus was cloned into pEarlygate202-YC to generate ATM-N-YFP^C^. *Agrobacterium* GV3101 cells containing each gene were cultured overnight, and then adjusted the OD_600_ at 0.6–0.8 using 10 mM MgCl_2_. An equal volume of each culture was mixed together for injection. At 2 days after injection, YFP fluorescence was detected with a Leica SP8 confocal microscope.

### Pull-down assays

*RUG3* was cloned into pGEX-4T-1 at the *Sa1*I-*Bam*HI sites to generate *GST-RUG3* fusion protein, while the *ATM* N-terminus (ATM-N, 1-900 bp) was cloned into pMAL-c2X at the *Eco*RI-*Bam*HI sites to generate MBP-ATM-N. GST- or MBP-tagged proteins were expressed in *Escherichia coli* BL21 cells. For the pull-down assays, equal amounts of purified MBP-ATM-N were added to tubes containing GSH-Sepharose resin (L008; TransGen Biotech Co., Ltd.) plus GST or GST-RUG3 protein, respectively. Anti-MBP antibodies (E8030; New England Biolabs, Ipswich, MA) were used to detect the interaction between RUG3 and ATM-N.

### Statistical analysis

All data were analyzed using statistical software SPSS 13.0. Statistical significance was determined using a one-way ANOVA analysis of variance (ANOVA, Duncan tests). In all Figures, statically significant differences are marked with ‘*’(*P* < 0.05), ‘**’(*P* < 0.01), ‘***’(*P* < 0.001) or ‘ns’ (no significance; *P* > 0.05). Moreover, different letters indicate a significant difference at *P* < 0.05.

## Additional Information

**Accession codes:** Sequences of the genes in this paper may be found in the TAIR database under the gene ID: AT5G60870 (RUG3), AT3G48190 (ATM).

**How to cite this article:** Su, C. *et al*. RUG3 and ATM synergistically regulate the alternative splicing of mitochondrial *nad2* and the DNA damage response in *Arabidopsis thaliana. Sci. Rep.* **7**, 43897; doi: 10.1038/srep43897 (2017).

**Publisher's note:** Springer Nature remains neutral with regard to jurisdictional claims in published maps and institutional affiliations.

## Supplementary Material

Supplemental Materials
