# Supplementary material for: RUG3 and ATM synergistically regulate the alternative splicing of mitochondrial nad2 and the DNA damage response in Arabidopsis thaliana
Source: Sci Rep. 2017 Mar 6;7:43897. doi: 10.1038/srep43897 (PMC5338318; doi:10.1038/srep43897)

Title

RUG3 and ATM synergistically regulate the alternative splicing of mitochondrial *nad2* and the DNA damage response in *Arabidopsis thaliana*

Authors and addresses

Chao Su[1,2,3¶], Hongtao Zhao[2,4¶], Yankun Zhao[2,5¶], Hongtao Ji[1], Youning Wang[1], Liya Zhi[2], Xia Li[1*]

[1]State Key Laboratory of Agricultural Microbiology, College of Plant Science and Technology Huazhong Agricultural University. Wuhan 430070, P.R. China

[2]Center for Agricultural Research Resources, Institute of Genetics and Developmental Biology, Chinese Academy of Sciences. Hebei 050021, P.R. China

[3]University of Chinese Academy of Sciences, Beijing 100049, P.R. China

[4]College of Life Sciences, Hebei Normal University, Hebei 050024, P.R. China

[5]Shijiazhuang Academy of Agricultural and Forestry Sciences, Hebei 050041, P.R. China

¶ Those authors contributed equally to this work.

Corresponding author

Xia Li,

Ph.D., Professor

State Key Laboratory of Agricultural Microbiology, College of Plant Science and Technology, Huazhong Agricultural University. Wuhan 430070, P.R. China

E-mail: xli@mail.hzau.edu.cn

Telephone number: +86-311-85871744

E-mail address of each author

Chao Su: chao.su@biologie.uni-freiburg.de

Hongtao Zhao: zhaohongtao325@126.com

Yankun Zhao: 20617352@qq.com

Hongtao Ji: htji@mail.hzau.edu.cn

Youning Wang: youningwang@mail.hzau.edu.cn

Liya Zhi: 574633276@qq.com

Xia Li: xli@mail.hzau.edu.cn

Supplementary materials

The following materials are available in the online version of this article.

Supplementary Fig. S1 The T-DNA insertion analysis for the *rug3-1* and *rug3-2* mutants.

Supplementary Fig. S2 The *rug3* mutant shows developmental retardation.
Supplementary Fig. S3 Phenotypic analysis of the *rug3* mutant in whole life.
Supplementary Fig. S4 *RUG3* overexpression rescues the developmental retardation of *rug3-1*.
Supplementary Fig. S5 qRT-PCR analysis of the expression of cell cycle related genes.
Supplementary Fig. S6 Root phenotypic analyses of WT and *rug3-1* plants treated with HU.
Supplementary Fig. S7 Phenotypic analyses of WT, *rug3-1* and *Re-4* under MMS treatment.
Supplementary Fig. S8 Analysis of RUG3 GEF activity and interactions with RAN.
Supplementary Fig. S9 Subcellular localization of ATM N-terminal.
Supplementary Fig. S10 Genetic analysis of *RUG3* and *ATM*.
Supplementary Fig. S11 ROS accumulation in *rug3-1*.
Supplementary Fig. S12 *RUG3* and *ATM* responded to H_2O_2 synergistically.
Supplementary Fig. S13 qRT-PCR analysis of the expression of *RAD51* and *BRCA1*.
Supplementary Table S1 Sequences of the primers used in this study.
The full-length gel for Fig. 1b.
The full-length gel for Fig. 2a.
The full-length gel for Fig. 4a.
The full-length gel for Fig. 5c.
The full-length gel for Fig. 6d.

Supplementary Fig. S1

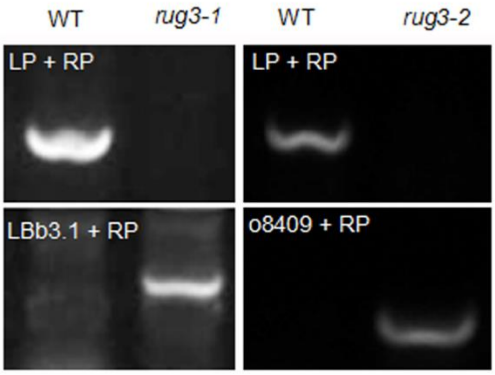

Supplementary Fig. S1 The T-DNA insertion analysis for the *rug3-1* and *rug3-2* mutants. LP and RP are the primers use to detect the *RUG3* gene, LBb1.3 is the primer on the insert fragment of *rug3*-1 and o8409 is the primer on the insert fragment of *rug3-2*.

Supplementary Fig. S2

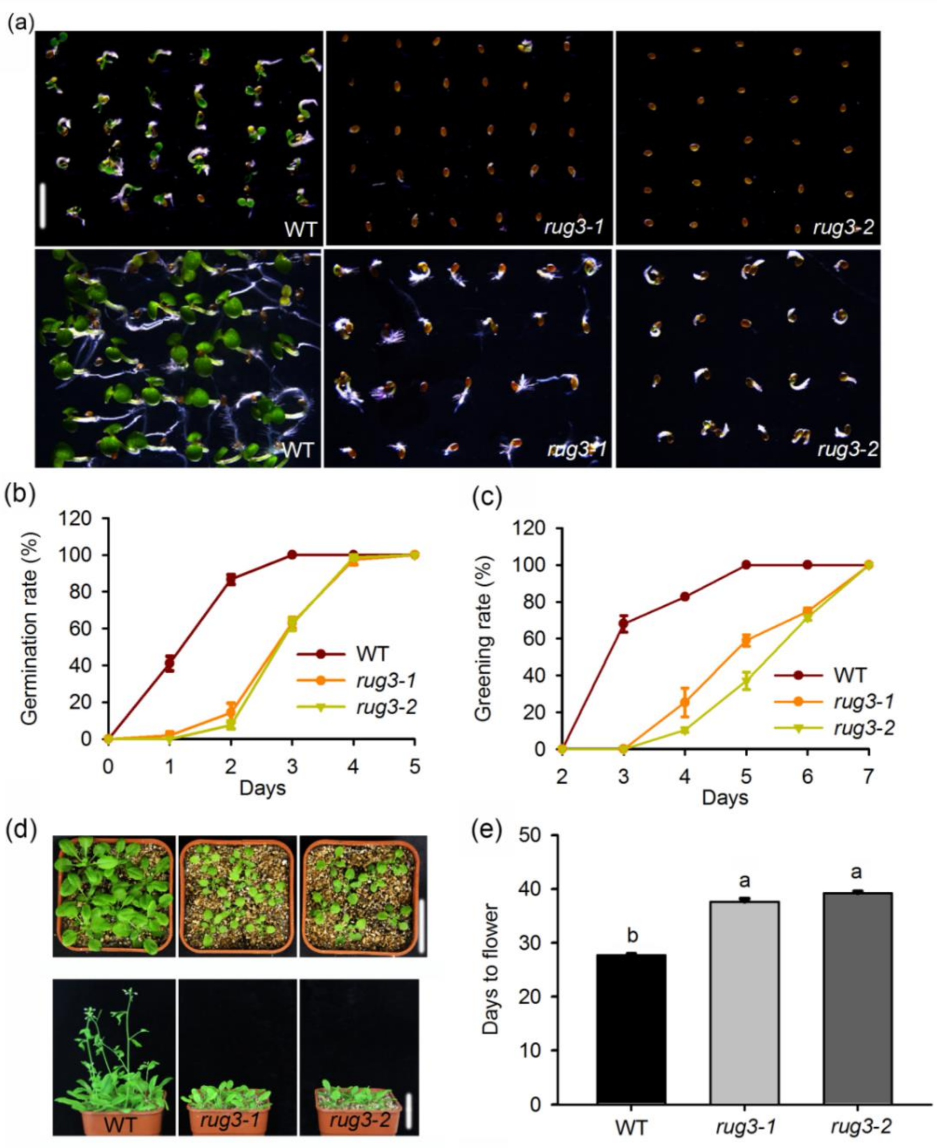

Supplementary Fig. S2 The *rug3* mutant shows developmental retardation. (a) Phenotypic analysis of WT, *rug3-1* and *rug3-2* plants grown on MS medium. The images were taken at 3 DAG (top) and 8 DAG (bottom), respectively, Bar = 6 mm. (b and c) Quantitative analysis of the germination rates (b) and greening rates (c) of the WT, *rug3-1* and *rug3-2* plants shown in (a). Three biological repeats were done with similar results. The data shown are means $\pm$ SD. (d) The growth of *rug3-1* and *rug3-2* seedlings was retarded compared with WT seedlings. Nine-day-old seedlings grown on MS medium were transferred to soil for 15 days (top) or 30 days (bottom) and then photographed, Bar = 4 cm. (e) Quantitative analysis of the flowering time of WT, *rug3-1* and *rug3-2* plants. Three biological repeats were done with similar results. The data shown are means $\pm$ SD. Different letters represent significant differences (Student-Newman-Kuels test, $P < 0.05$), Bar = 4 cm.

Supplementary Fig. S3

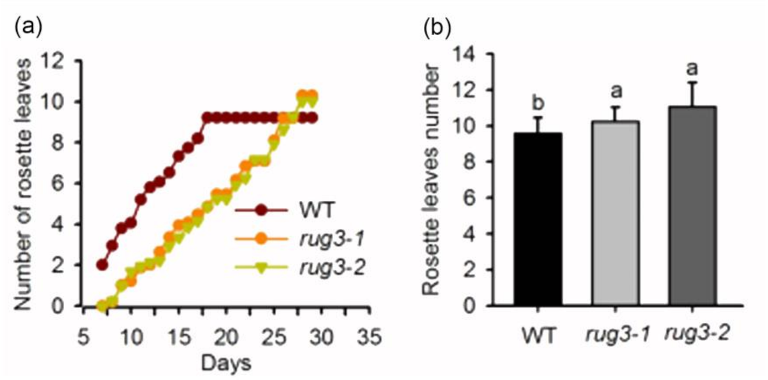

Supplementary Fig. S3 Analysis of the *RUG3* mutant life cycle. (a) Quantitative analysis of leaf initiation of different genotypes. (b) Quantitative analysis of the rosette leaf number at 28 DAG. Three biological repeats were done with similar results. The data shown are means + SD (n = 30). Different letters represent significant differences. Different letters represent significant differences (Student-Newman-Kuels test, $P< 0.05$)

Supplementary Fig. S4

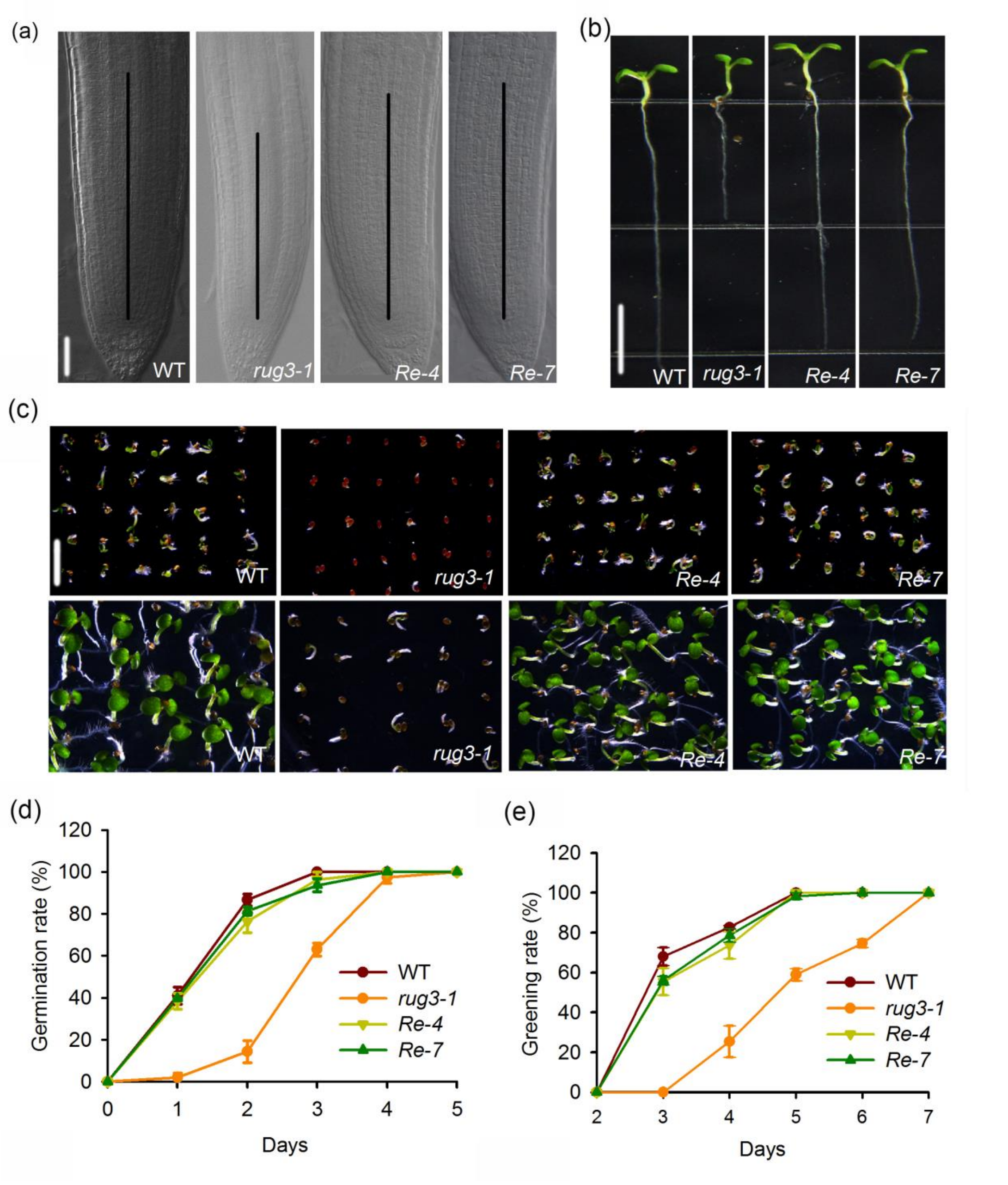

Supplementary Fig. S4 *RUG3* overexpression rescues the developmental retardation of *rug3-1*. (a and b) Root lengths of WT, *rug3-1* plants and two transgenic lines (*Re-4* and *Re-7*) grown on MS medium. The pictures were taken at 7 DAG. The vertical lines in (a) indicate the root meristem length. Bar=100 µm (a); Bar = 6 mm (b). (c) Phenotypic analysis of WT, *rug3-1* plants and the two transgenic lines grown on MS medium. The pictures were taken at 3 DAG (top) and 8 DAG (bottom), respectively. Bar = 6 mm. (d and e) Quantitative analysis of the germination rates (d) and greening rates (e) of WT, *rug3-1* plants and the two transgenic lines in (c). Three biological replicates were done with similar results. The data shown are means ± SD.

Supplementary Fig. S5

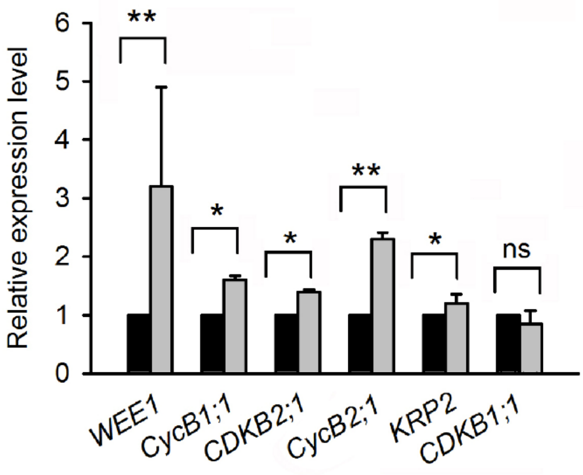

Supplementary Fig. S5 qRT-PCR analysis of the expression of cell cycle related genes. The expression of *CycB1;1*, *WEE1*, and *CDKB2;1*, *ACT2* was used as an internal control. Ten-day-old seedlings grown on MS medium were used for RNA extraction. Three biological replicates were done with similar results. The data shown are means ± SD. (Student's *t*-test, where *****P<0.05; ******P<0.01; ns indicates no significant difference). Black and grey bars indicate WT and *rug3-1* respectively.

Supplementary Fig. S6

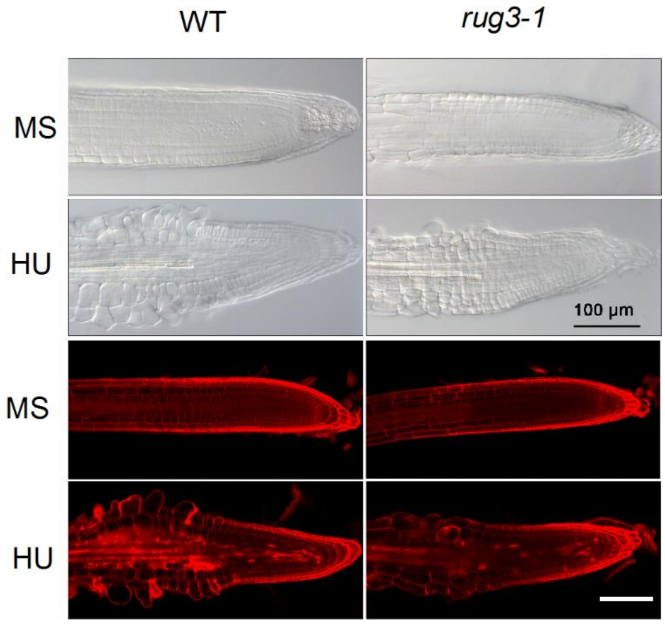

Supplementary Fig. S6 Root phenotypic analyses of WT and *rug3-1* plants treated with HU. Five-day-old seedlings grown on MS medium were transferred to MS medium containing 0 or 10 mM HU for another 24 h before being photographed. Lower panels were photographed after PI staining. Root tip samples were cut and immersed in 10 μM PI for 1 min and then washed three times with phosphate-buffered saline, a 543 nm HeNe laser was used for image acquisition (Leica SP8 confocal microscope) bar = 100 μm.

Supplementary Fig. S7

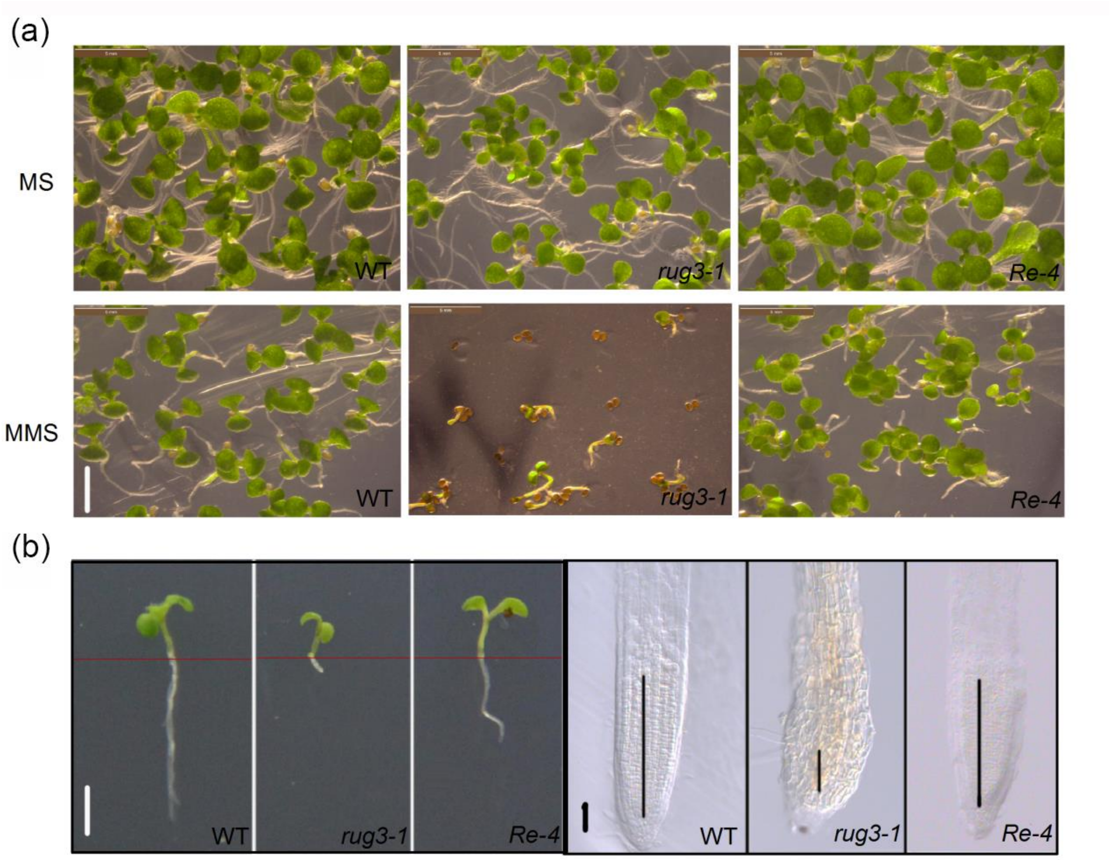

Supplementary Fig. S7 Phenotypic analyses of WT, *rug3-1* and *Re-4* under MMS treatment. (a) Phenotypic analysis of WT, *rug3-1* and *Re-4* plants grown on MS medium containing 0 or 0.01% MMS. The pictures were taken at 15 DAG. Bar= 4 mm. (b) Root lengths (left, Bar = 4 mm) and root meristem size (right, Bar = 100 µm) of WT, *rug3-1* and *Re-4* plants were analyzed from (a lower panel). The vertical lines in (b, right) indicate the root meristem length.

Supplementary Fig. S8

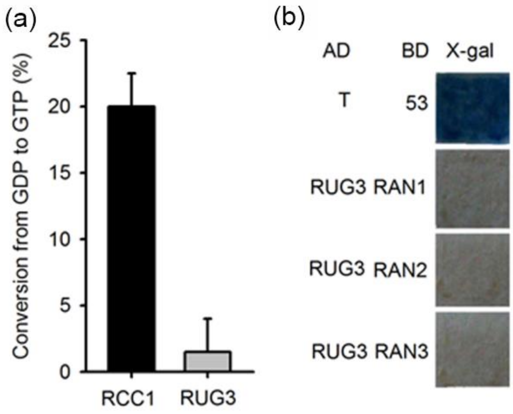

Supplementary Fig. S8 Analysis of RUG3 GEF activity and interactions with RAN. (a) GEF activity assay of RUG3. The Ran-GEF activity of *E. coli*-expressed GST-RCC1 and GST-RUG3 is shown as the percentage of [^{3}H]-GDP remaining at the end of the GEF assay. (b) Yeast two-hybrid analysis of the interaction between RUG3 and RANs using yeast co-transformant strains carrying both AD-RUG3 and BD-RAN (1/2/3), AD-T/BD-53 as a positive control.

Supplementary Fig. S9

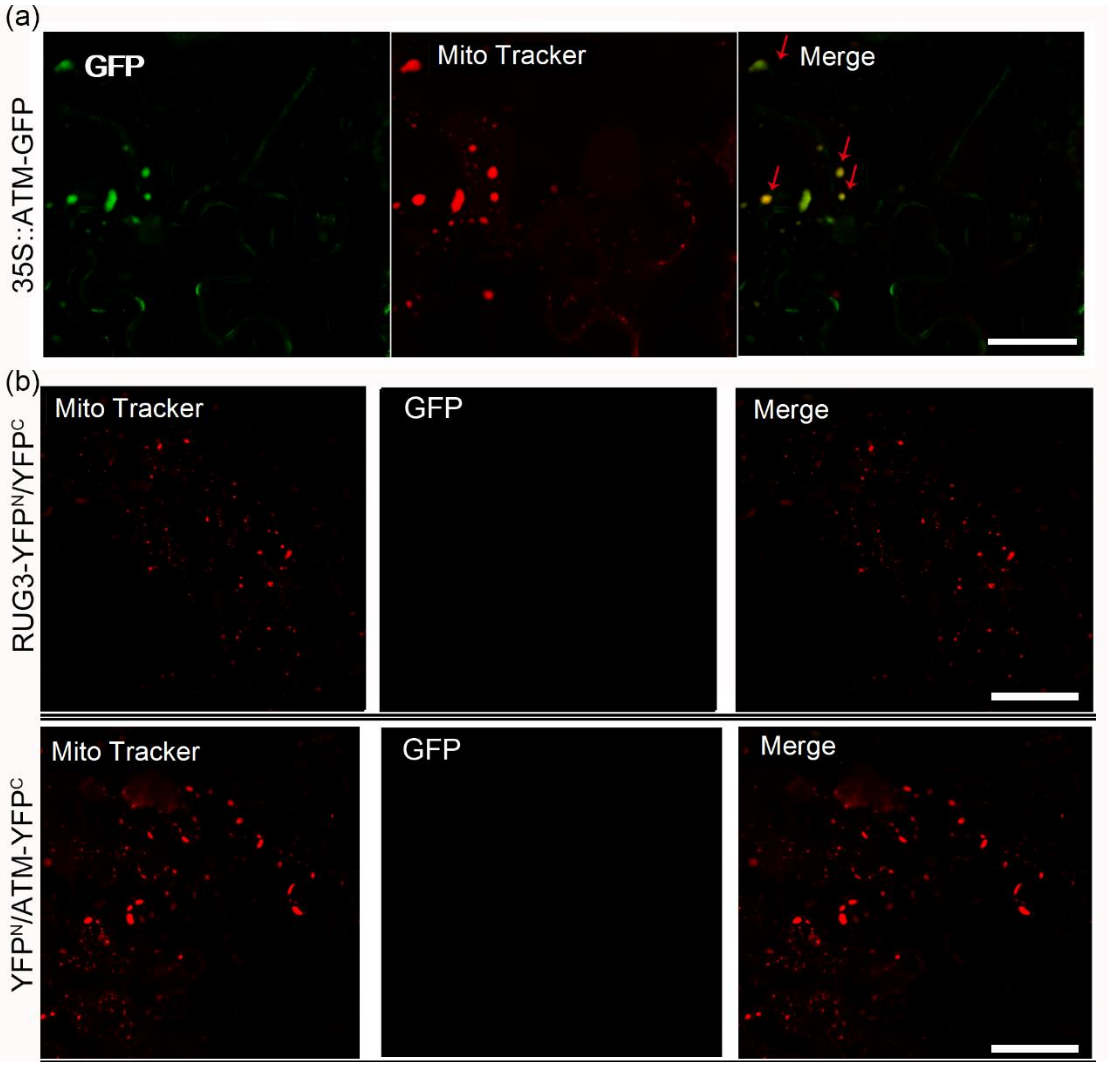

Supplementary Fig. S9 Subcellular localization of ATM N-terminal. (a) Construct harboring 35S::ATM-GFP was transformed into *N. benthamiana* leaves; ATM-GFP fluorescence was observed after 2 days of cultivation (Bar = 25 μm). (b) The negative control for the interaction between RUG3 and ATM in a BiFC assay. Constructs harboring RUG3-YN/YC and ATM-N-YC/YN were transformed into *N. benthamiana* leaves and YFP fluorescence was observed in the epidermal cells after 2 days of cultivation (Bar = 50 μm). The mitochondrial localization of the interaction was confirmed by MitoTracker staining.

Supplementary Fig. S10

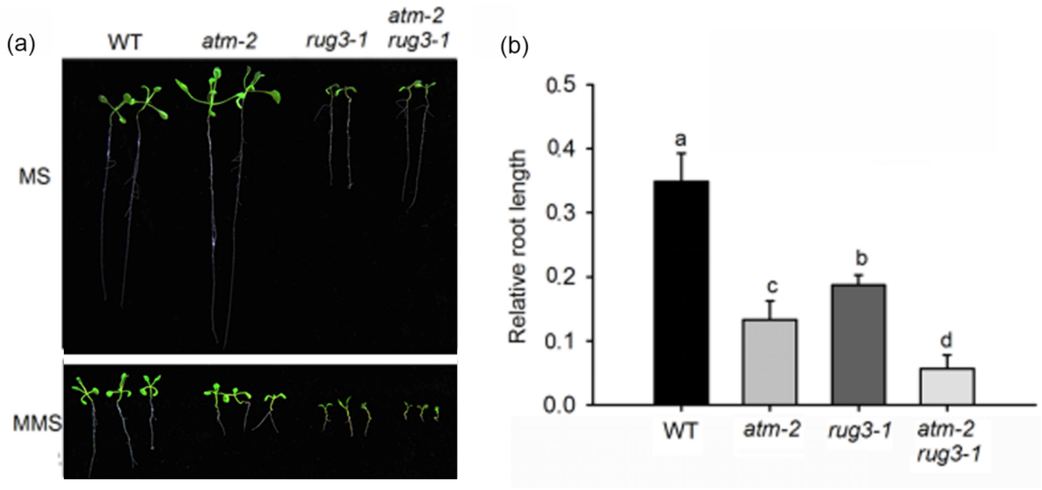

Supplementary Fig. S10 Genetic analysis of *RUG3* and *ATM*. (a) Phenotypic analysis of WT, *rug3-1*, *atm-2*, and *rug3-1atm-2* plants grown vertically on MS medium containing 0 or 0.01% MMS. The pictures were taken at 25 DAG. (b) Quantitative analysis of the relative root length (Root growth is expressed relative to that of the plants in MS medium without MMS) in plants grown vertically on MS medium containing 0 or 0.01% MMS. Three biological replicates were done with similar results. The data shown are means ± SD. Different letters represent significant differences (Student-Newman-Kuels test, $P < 0.05$). Bar=1 cm.

Supplementary Fig. S11

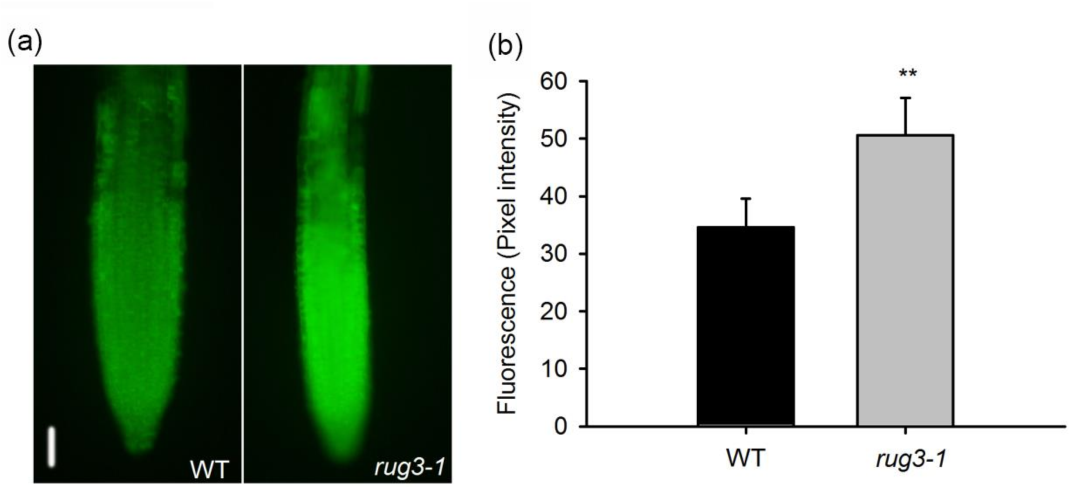

Supplementary Fig. S11 ROS accumulation in *rug3-1*. (a) Analysis of the H_2O_2 level in the primary root tips of WT and *rug3-1* plants by DCFH-DA staining at 7 DAG. Bar=100 μm. (b) Quantitative analysis of the DCFH-DA staining intensity in (a) with ImageJ software. Three biological replicates were done with similar results. The data shown are means + SD (n = 30). (Student's *t*-test, where ***P*<0.01).

Supplementary Fig. S12

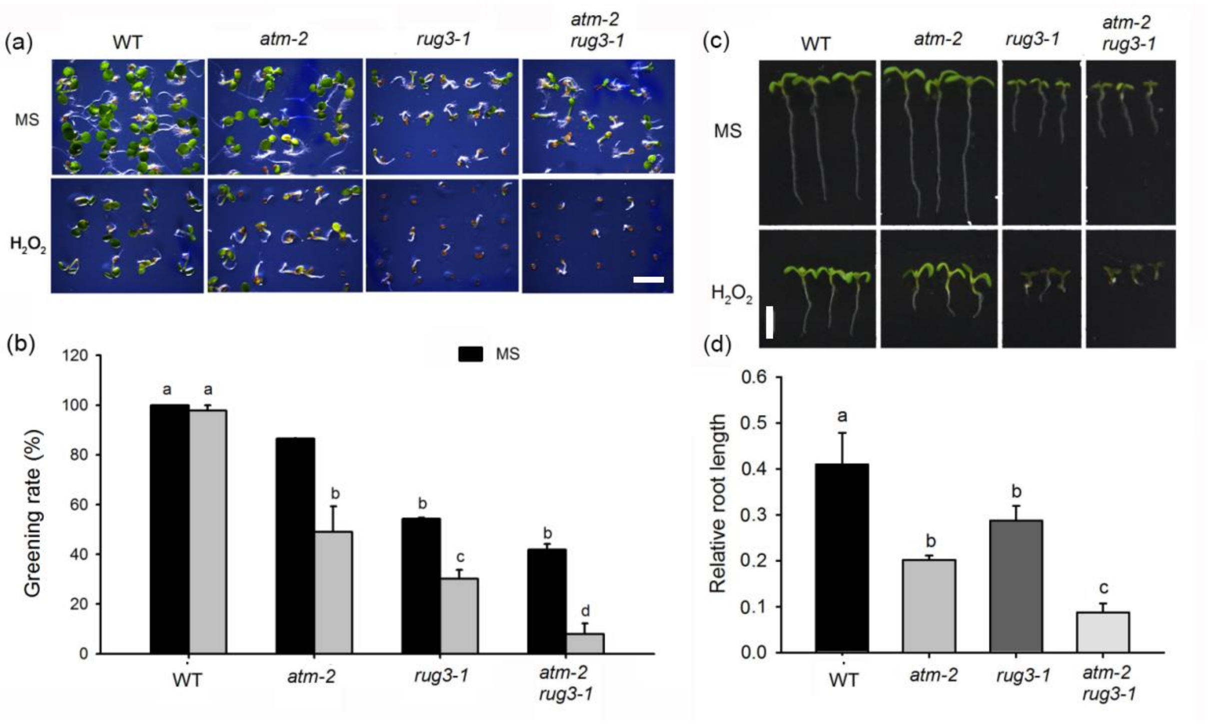

Supplementary Fig. S12 *RUG3* and *ATM* responded to H_2O_2 synergistically. **(**a) Phenotypic analysis of WT, *rug3-1*, *atm-2*, and *rug3-1atm-2* plants grown on MS medium containing 0 or 2 mM H_2O_2. The pictures were taken at 4 DAG. Bar = 6 mm. (b) Quantitative analysis of the greening rate on MS medium containing 0 or 2 mM H_2O_2 at 5 DAG. Three biological replicates were done with similar results. The data shown are means $\pm$ SD. Different letters represent significant differences (Student-Newman-Kuels test, $P<0.05$). (c) Phenotypic analysis of root length. The images were taken at 5 DAG. (d) Relative root length (Root growth is expressed relative to that of the plants in MS medium without H_2O_2) analysis of WT, *rug3-1*, *atm-2*, and *rug3-1atm-2* plants grown on MS medium containing 0 or 2 mM H_2O_2. Bar=6 mm. Three biological replicates were done with similar results. The data shown are means $\pm$ SD. Different letters represent significant differences (Student-Newman-Kuels test, $P<0.05$).

Supplementary Fig. S13

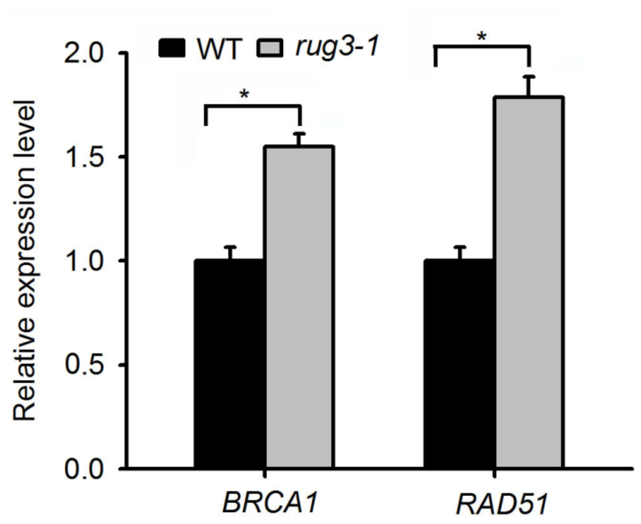

Supplementary Fig. S13 qRT-PCR analysis of the expression of *RAD51* and *BRCA1*. *ACT2* was used as an internal control. Ten-day-old seedlings grown on MS medium were used for RNA extraction. Three biological replicates were done with similar results. The data shown are means ± SD. (Student's *t*-test, where ***P**<0.05).

Supplementary Table S1 Sequences of the primers used in this study.

Name	Sequence	Gene	Purpose
RUG3-GUS-F<br>RUG3-GUS-R	CGGGATCCCAGATTAGGAGTAGAGTGGATG<br>GGAATTCGAGTTTCATCACTACAGTCTAC	*RUG3pro*	*RUG3pro::GUS* construct
RUG3-GFP-F<br>RUG3-GFP-R	GCGTCGACATGGCAGCGTTAAGCCA<br>CGGGATCCTCAAGGTGATCTTGAGACTAAAC	*RUG3*	*RUG3pro::GFP* construct
RUG3-GST-F<br>RUG3-GST-R	CGGGATCCATGGCAGCGTTAAGCCAC<br>GCGTCGACTCAAGGTGATCTTGAGACTAAAC	*RUG3*	*RUG3-GST* construct
ATM-MBP-F<br>ATM-MBP-R	CGGGATCC ATGAAATTACAAAACCCAGATAAG<br>GCTCTAGA TTAGGATGGAGAACCAATGAACTTAC	*ATM*	*ATM-MBP* construct
KRP2-RT-F<br>KRP2-RT-R	AGGAGAAGAGAACGAGATGTG<br>CAGCCACCGAAGAAGAAT	*KRP2*	RT-PCR
CycB2;1-RT-F<br>CycB2;1-RT-R	CAGTTCCAAGTGCTAACGACT<br>CGCTTTGTCCGAAATGAG	*CycB2;1*	RT-PCR
CDKB1;1-RT-F<br>CDKB1;1-RT-R	GACCTAATCCTAAGCCTCTTGA<br>TGCTGCTCAGTTGGTGTTC	*CDKB1;1*	RT-PCR
CDKB2;1-RT-F<br>CDKB2;1-RT-R	GAAGGGACATACGGGAAAG<br>CACCAAGAAGAACCTCTGGA	*CDKB2;1*	RT-PCR
CycB1;1-RT-F<br>CycB1;1-RT-R	CTGGGAAACCAAAGGTAGTAGA<br>AAGAGCACTGAGACCAACAAG	*CycB1;1*	RT-PCR
WEE1-RT-F<br>WEE1-RT-R	AAGATGTCGCCAGGAGAGT<br>AACCCAAGAGCAGCAAGA	*WEE1*	RT-PCR
CycB1;1-Q-F<br>CycB1;1-Q-R	CTCAAAATCCCACGCTTCTTGTGG<br>CACGTCTACTACCTTTGGTTTCCC	*CycB1;1*	Real Time Q - PCR
WEE1-Q-F<br>WEE1-Q-R	TGGTGCTGGACATTTCAGTCGG<br>CAAGAGCTTGCACTTCCATCATAG	*WEE1*	Real Time Q-PCR
NAD2-intro3F<br>NAD2-intro3R	CACTGCAGTTCCTTTTCGGG<br>TACCACCCACCCTACCCTAC	*NAD2*	Real Time Q-PCR
CDKB2;1-Q-F<br>CDKB2;1-Q-R	CCGGGAAAATCGTCGCTCTA<br>GGAGAGTGGTGGAAGGAACG	*CDKB2;1*	Real Time Q-PCR
RAD51-Q-F<br>RAD51-Q-R	CGAGGAAGGATCTCTTGCAG<br>GCACTAGTGAACCCCAGAGG	*RAD51*	Real Time Q-PCR

BRCA1-Q-F<br>BRCA1-Q-R	CCATGTATTTTGCAATGCGTG<br>TGTGGAGCACCTCGAATCTCT	*BRCA1*	Real Time Q-PCR
RUG3-F<br>RUG3-R	ATGGCAGCGTTAAGCCAC<br>TTAGGATGGAGAACCAATGAACTTAC	*RUG3*	*RUG3* expression
RUG3-Q-F<br>RUG3-Q-R	GCAAAATCTCTGCCATTGCTAC<br>CATCTCCTTCTTCTCTTCCCCAC	*RUG3*	Real Time Q-PCR
UBC-F<br>UBC-R	CTGCGACTCAG^GGAATCTTCTAA<br>TTGTGCCATTGAATTGAACCC	*UBC*	RT-PCR<br>Real Time Q-PCR
P1<br>P2<br>P3	CCACTTCGATCAATTAGCCAAG<br>ATGCTATGGTCTCAATGCCCTT<br>ATAGAAGCAATGCTGCAGAAAAAG	*nad2*	RT-PCR
ACT2-F<br>ACT2-R	CTTGCACCAAGCAGCATGAA<br>CCGATCCAGACACTGTACTTCCTT	*ACT2*	RT-PCR
35S-RUG3-F<br>35S-RUG3-R	GCTCTAGAATGGCAGCGTTAAGCCAC<br>CGGGATCCTCAAGGTGATCTTGAGACTAAAC	*RUG3*	*35S::RUG3-GFP* and *RUG3pro::RUG3-GFP* construct
Pro-RUG3-F<br>Pro-RUG3-R	AACTGCAGCAGATTAGGAGTAGAGTGGATG<br>GCTCTAGAGAGTTTCATCACTACAGTCTAC	*RUG3* Promoter	*RUG3pro::RUG3-GFP* construct
CycB2;1-Q-F	AAGTGTGAGGTCTTGGCGTC	*CycB2;1*	Real Time Q-PCR
CycB2;1-Q-R	ACTCACCAGCTTCCGTGAAC		
CDKB1;1-Q-F	TGAGATGGTTCGGAGGCAAG	*CDKB1;1*	Real Time Q-PCR
CDKB1;1-Q-R	CAGTCACGCAGTGTGGAAAC		
KRP2-Q-F	CGACGGTGAAACGAAGGAAG	*KRP2*	Real Time Q-PCR
KRP2-Q-R	CCTGCTGAATTTCTCGCCAC		
ATM-N-F	GGGGACAAGTTTGTACAAAAAAGCAGGCTTC<br>ATGAAATTACAAAACCCAGATAAGAAAAC	*ATM*	ATM-GFP
ATM-N-R	GGGGACCACTTTGTACAAGAAAGCTGGGTCT<br>CTTGATAAACTATCCCCGGATTTC		
LP	GATGTATTCACTTGGTAAAGCTCTAG	*RUG3*	*T-DNA insertion analysis for the rug3-1 and rug3-2*
RP	GGTGATCTTGAGACTAAACACAGAGC		
LBb1.3	ATTTTGCCGATTTCGGAAC		
o8409	ATATTGACCATCATACTCATTGC		

The full-length gel for Fig. 1b

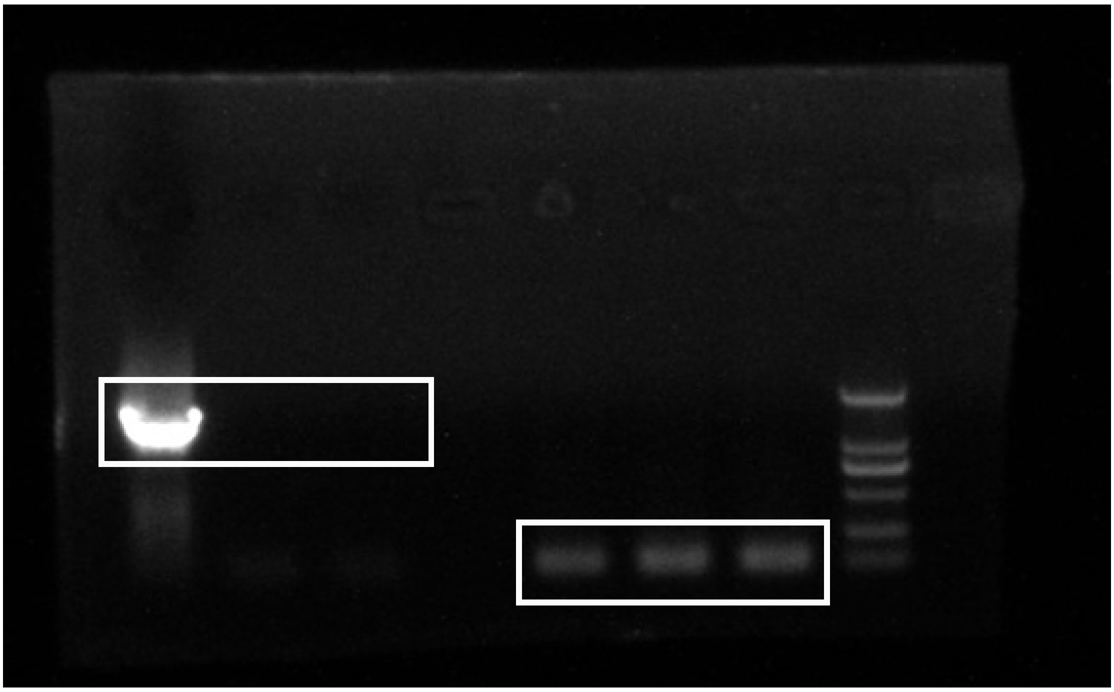

The full-length gel for Fig. 2a

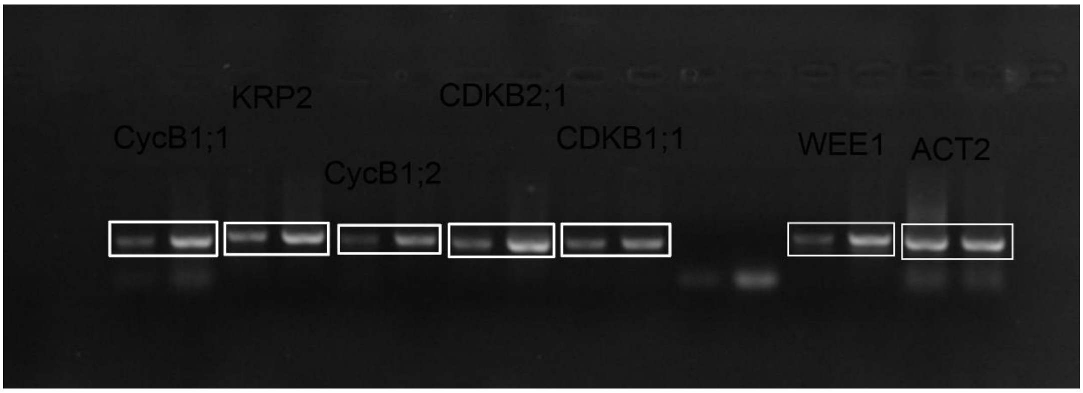

The full-length gel for Fig. 4a

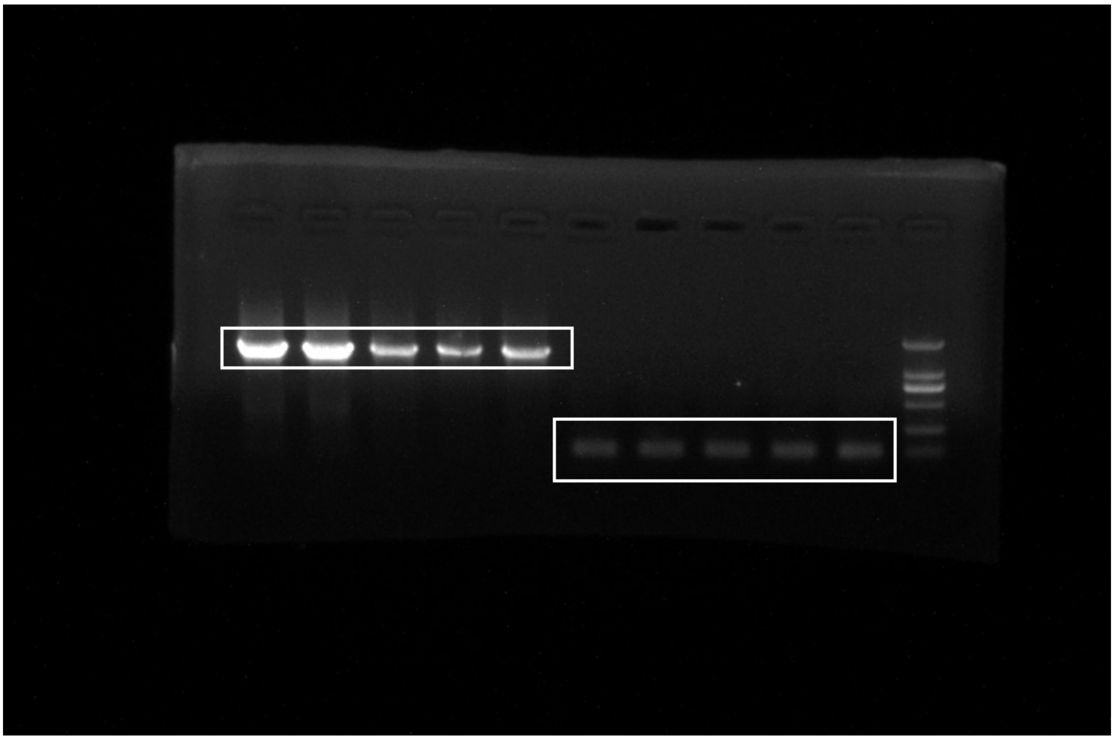

The full-length gel for Fig. 5c

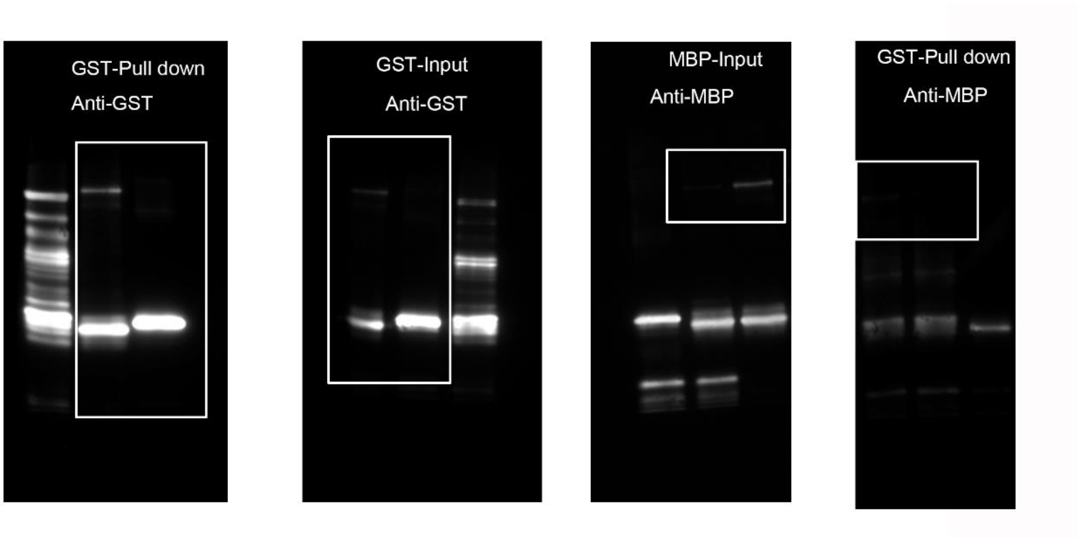

The full-length gel for Fig. 6d

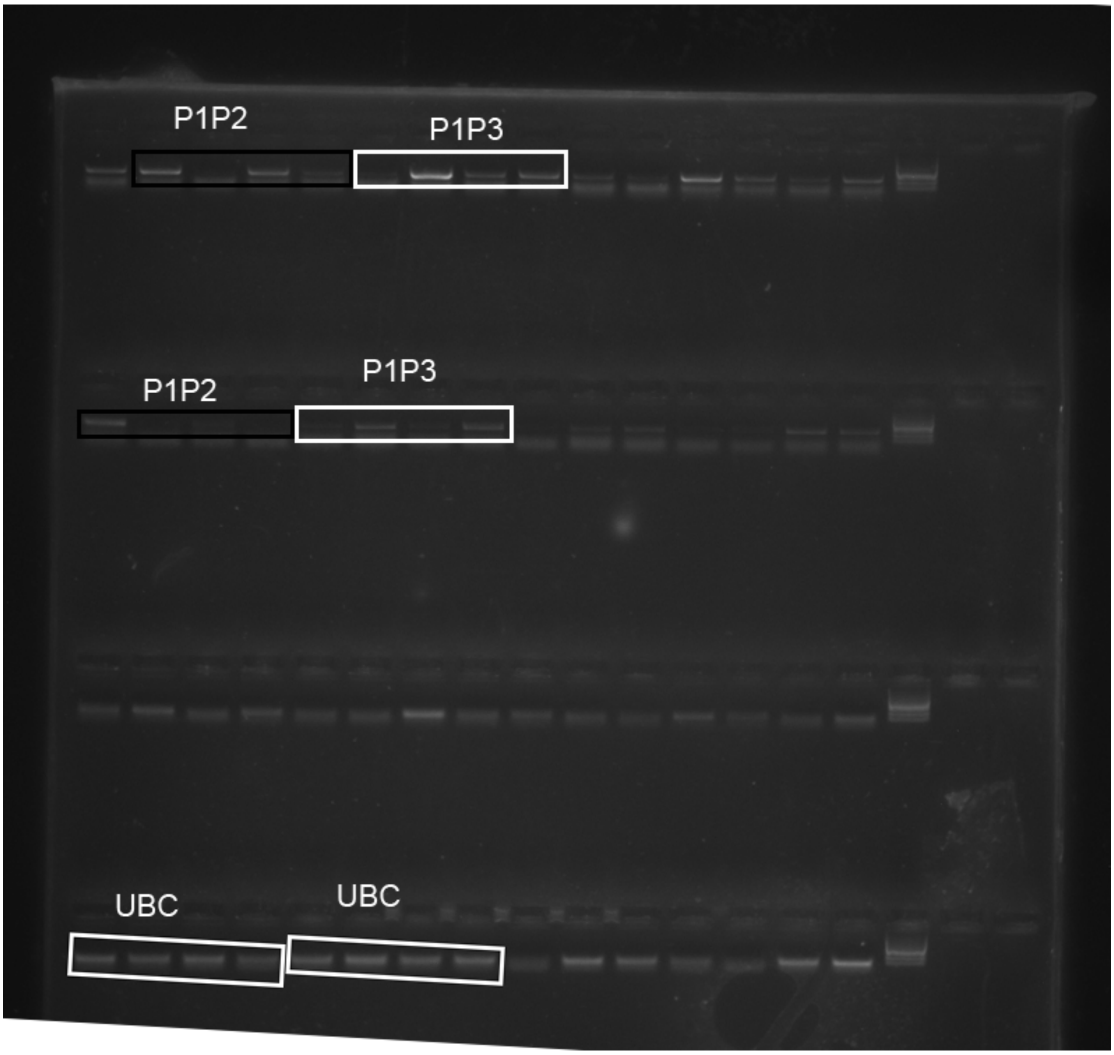

Supplement: Supplemental Materials [file srep43897-s1.pdf]
